# Soft Interferometric Nanostrain Sensor Reveals Solid‐Liquid Interfacial Tension Oscillation Amplified by Competitive Adsorption

**DOI:** 10.1002/smll.202508858

**Published:** 2025-12-08

**Authors:** Samuel K.S. Cheng, Maryam Jalali‐Mousavi, Jian Sheng

**Affiliations:** ^1^ College of Engineering and Computer Science Texas A&M University‐Corpus Christi Corpus Christi TX 78412 USA

**Keywords:** competitive adsorption, digital holographic interferometry, fetal bovine serum, interferometric nanostrain sensor, solid‐liquid interfacial tension oscillation

## Abstract

Understanding the adsorption of protein mixtures is significant for engineering biosurface functionalities that affect biomaterial hemocompatibility and infections over medical instruments. However, existing methods cannot detect real‐time competitive adsorption events or require fluorophore labelling that may alter the adsorption characteristics. An interferometric nanostrain sensor has been developed to investigate real‐time adsorption of unlabeled proteins. The sensor exploits the elastocapillary effect on the substrate nanometer deformation by a sessile protein drop to measure instantaneous interfacial tensions at its three‐phase contact line. Using fetal bovine serum (FBS), it is shown that asymptotically, the solid‐liquid interfacial tension (γ_*SL*_) decreases with increasing FBS concentrations, and the solid‐vapor tension (γ_*SV*_) remains uncorrelated. Results link molecular adsorption events with macroscale surface energy. Dynamically, γ_*SL*_ shows Langmuir adsorption characteristics on a coarse timescale. With the sensor's high spatiotemporal resolutions (2.38 nm or <0.25 mN m^−1^), it is reported for the first time a high‐frequency, low‐amplitude oscillatory modulation of γ_*SL*_ at a fine timescale over the Langmuir adsorption curve. Compared with other colloids with fewer competing elements of adsorption, this oscillation is intrinsic to any colloidal system but strongly amplified by competitive adsorption. The sensor provides a novel ensemble‐averaging technology to quantify competitive adsorption, thereby revealing new mechanisms for protein‐surface interactions.

## Graphical abstract

An interferometric nanostrain sensor is developed to investigate unlabeled protein adsorption by directly measuring real‐time interfacial tensions at unprecedented resolution (<0.25 mN m^−1^). A high‐frequency, low‐amplitude oscillation of the interfacial tension is found to be superimposed over the Langmuir adsorption. While this oscillation is intrinsic to any colloidal system, its amplitude is found to be regulated by competitive adsorption.

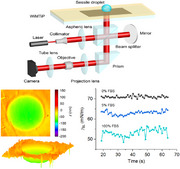

## 1 Introduction

Adsorption of proteins onto a solid substrate is ubiquitous but complex: common because it often constitutes the first stage of many biological processes, including biofilm formation^[^ ^1^ ^]^ and the blood coagulation cascade,^[^ ^2^ ^]^ while complex owing to many protein‐specific mechanisms, such as molecular structures,^[^ ^3^ ^]^ conformational changes,^[^ ^4^ ^]^ and non‐Langmuir adsorption^[^ ^5^ ^]^ that differs from inert nanoparticles’ stochastic kinetics.^[^ ^6^ ^]^ In the presence of multiple proteins, competitive adsorption leads to a changing protein film composition that differs at the early and late stages of adsorption,^[^ ^7^ ^]^ which is governed by proteins’ size, mobility, attachment affinity, and surface properties.^[^ ^8^ ^]^ Smaller proteins with higher diffusivity reach the surface first and are adsorbed, which is subsequently replaced by larger molecular weight proteins with lower mobility but higher adsorption affinity.^[^ ^9^ ^]^ This competitive adsorption mechanism is important for blood‐contacting biomaterials, as the adsorbed protein layers define the functionalities.^[^ ^10^, ^11^ ^]^ Understanding of the underlying competitive adsorption process and its kinetics will lead to a better prediction of the protein film composition and biomaterials’ response to blood.^[^ ^3^ ^]^

Existing methods to investigate competitive protein adsorption include ensemble‐averaging and single‐molecular tracking techniques. The former, comprising surface plasmon resonance (SPR) and quartz crystal microbalance (QCM), measures the change in the optical^[^ ^12^ ^]^ and acoustical^[^ ^13^ ^]^ resonance, respectively, when proteins adsorb onto the surface. In the context of competitive adsorption, a mixture of proteins will produce only a single ensemble‐averaged measurement, which requires additional sequential single‐protein adsorption experiments to decipher the adsorption process,^[^ ^7^, ^14^, ^15^, ^16^ ^]^ thus prohibiting real‐time observations of interfacial dynamics induced by complex protein mixtures.^[^ ^17^ ^]^ Furthermore, the need for a thin substrate (1 Å – 1 µm) for optimal signal limits the applications of thicker substrates.^[^ ^18^ ^]^ Single‐molecular tracking approaches utilize total internal reflection fluorescence microscopy (TIRFM) to track individual adsorption events of proteins labeled with fluorophores.^[^ ^19^ ^]^ Since TIRFM only excites surface‐adsorbed labeled proteins directly, it can resolve protein kinetics at high spatiotemporal resolutions.^[^ ^20^ ^]^ Protein specificity is achieved by tagging different fluorophores to each species.^[^ ^21^, ^22^, ^23^ ^]^ However, concerns about proteins’ properties altered by labelling fluorophores and the limited applicability to very low concentrations^[^ ^17^, ^24^ ^]^ remain.

In this study, a first‐of‐its‐kind soft interferometric nanostrain sensor is developed to investigate protein adsorption/desorption kinetics by directly measuring the solid‐liquid interfacial tension γ_*SL*_, of unlabeled protein drops on soft substrates. The sensor measures the 3D substrate deformation caused by the drops at the three‐phase contact line (e.g., wetting ridge^[^ ^25^ ^]^) and subsequently uses the ridge's geometry to obtain γ_*SL*_.^[^ ^26^ ^]^ Using fetal bovine serum (FBS) as a model system, we demonstrate the method's capability in detecting real‐time adsorption events and relate microscopic kinematics to macroscopic interfacial stresses. When investigating the temporal γ_*SL*_ dynamics, we observed an unreported high‐frequency, low‐amplitude oscillation of γ_*SL*_ that is superimposed on a traditional Langmuir adsorption curve. Furthermore, we performed mechanistic studies on these oscillations with respect to timescales (short‐ & long‐term), FBS concentrations (0–100%), and solutions (e.g., FBS, bovine serum albumin (BSA), nanoparticles (NPs), denatured FBS, and FBS with ionic surfactants). Although this method adopts an ensemble‐averaging approach, it enables observations of adsorption occurring at molecular scales with direct macroscopic impacts on interfacial tensions.

## 2 Results

### 2.1 Interferometric Nanostrain Sensor System and Its Measurement Principle

**Figure 1:**
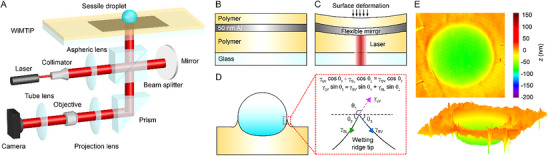
Experimental setup and working principle of digital holographic interferometry (DHI) & WiMTiP (wrinkle‐free nm metallic thin film in polymer) nano‐strain sensor. A) Optical setup: a soft WiMTiP strain sensor and a DHI. Working principle of a WiMTiP sensor B) with and C) without stresses. Red: an object beam illuminates and reflects by a WiMTiP sensor for encoding deformation. D) Elastocapillarity of a liquid drop over a soft substrate. Inset: Geometry of a wetting ridge and interfacial tension balance at its tip. E) Sample deformation map of a 0.4 µL water drop measured by the proposed sensor. Top: 2D deformation map, Bottom: 3D deformation profile.

The system (**Figure** **1**A) is comprised of a WiMTiP (wrinkle‐free metallic thin film in polymer)^[^ ^27^ ^]^ sensor (Figure 1B) to register nanometer (nm) scale deformations (e.g., 2.38 nm) and a digital holographic interferometer (DHI, Figure 1A and Section S.1, Supporting Information) to read them out optically. As elucidated in Figure 1B, a WiMTiP sensor is made of a nm (e.g., 50 nm) thick wrinkle‐free metal (e.g., *Al*) thin film embedded in a polymer (e.g., polydimethylsiloxane, PDMS) matrix over a transparent solid substrate (e.g., a glass slide). Although simple in principle, the successful synthesis of a WiMTiP composite has only been achieved recently by suppressing intrinsic metal‐polymer interfacial instability with nanoparticle jammers.^[^ ^27^ ^]^ Owing to its nm thickness and optical smoothness, the embedded film, as a flexible mirror, inherits the bulk mechanical properties of the polymer and deforms readily with the encasing polymer when stresses are applied (Figure 1C). The deformation registered as a phase change in the collimated object beam (Figure 1C) can then be optically read out by a DHI (Figure 1A) as interference fringes (Section S.2 and Figure S1, Supporting Information). The interferograms are decoded numerically to reconstruct 3D sensor deformations (Figure S1 and Section S.2, Supporting Information). By determining the noise floor, the sensor has achieved a z‐deformation uncertainty of 2.38 ± 0.51 nm with a field of view (FOV) of 1.5 mm × 1.5 mm or 3.00 ± 1.14 nm for a FOV of 2.6 mm × 2.6 mm (Section S.3 and Figure S2A, Supporting Information). Note that the FOV can be changed by altering the projection lens's magnification (Figure 1A and Section S.1, Supporting Information). Owing to its high *z* accuracy, nm deformation at the interface can be directly measured without the need for deformation enhancement by complex geometries.^[^ ^28^, ^29^, ^30^ ^]^ When compared to existing techniques to visualize surface deformation, such as laser scanning confocal microscopy (LSCM) and X‐ray microscopy, the proposed method is distinctively simpler and more robust in both temporal resolution (<0.1us) and measurement area (e.g., 1.5 × 1.5 mm), which differs greatly from LSCM (e.g., minutes^[^ ^31^ ^]^), and X‐ray microscopy (e.g., 13 × 13 µm^[^ ^32^, ^33^ ^]^). This method opens up the possibility of probing interfacial phenomena over a large area with high spatiotemporal resolution in real time.

Differing from past approaches such as SPR, QCM, and TIRFM, we quantify protein‐surface kinetics by directly measuring the instantaneous γ_*SL*_ between the protein suspension (*L*) and the WiMTiP sensor (*S*), since any protein adsorption or desorption alters γ_*SL*_.^[^ ^34^, ^35^ ^]^ To achieve such a measurement in real‐time, we exploit the elastocapillary effect of wetting on a soft substrate, i.e., the formation of a wetting ridge caused by the liquid‐vapor (V) tension γ_*LV*_ at the three‐phase contact line.^[^ ^36^ ^]^ Since these interfacial tensions are instantaneously balanced at the tip of the wetting ridge (Inset in Figure 1D), one can extract the instantaneous γ_*SL*_ and γ_*SV*_^[^ ^26^ ^]^ when γ_*LV*_, contact angle, and geometries of the wetting ridge are known. Note that the WiMTiP sensor has an elasticity of ≈2.0 MPa as determined by AFM nano‐indentation (Section S.4 and Figure S2B, Supporting Information). Uncertainty analysis (**Table** **1** and Section S.9, Supporting Information) shows that the accuracy of γ_*SL*_ and γ_*SV*_ reaches <0.25 mN m^−1^.

**Table 1:** Statistics and uncertainty of γ_*SL*_for different cases. Nomenclates: FBS ‐ fetal bovine serum. SDS – sodium dodecyl sulfate. NP – nanoparticle. BSA – bovine serum albumin. $\bar{\gamma_{SL}^{R}}$ – time mean of $\gamma_{SL}^{R}\left(t\right)$ over a short time window of [*t*_0_,*t*_0_ + Δ*t*], i.e., $\bar{\gamma_{SL}^{R}}=\frac{1}{\Delta t}\int_{t_{0}}^{t_{0}+\Delta t}\gamma_{SL}^{R}\left(t\right)dt$, where Δ*t* = 1 min in the current analysis. $\sigma_{\gamma_{SL}^{\prime }}$ – one standard deviation of $\gamma_{SL}^{\prime }\left(t\right)$ within the window. *E*γ_*SL*_ – measurement uncertainty of γ_*SL*_. Note that $\gamma_{SL}\left(t\right)=\gamma_{SL}^{R}\left(t\right)+\gamma_{SL}^{\prime }\left(t\right)$. Highlighted: conservative estimation of the measurement uncertainty or accuracy.

Case	$\bar{\gamma_{SL}^{R}}$ [mN/m]	$\sigma_{\gamma_{SL}^{\prime }}$[mN/m]	$E_{\gamma_{SL}}$ [mN/m, Eqn. S10, Supporting Information]	$\sigma_{\gamma_{SL}^{\prime }}/E_{\gamma_{SL}}$
1% FBS, *t*_0_ = 1 min	65.11	0.57	0.02	28.5
25% FBS, *t*_0_ = 1 min	58.15	0.92	0.05	18.4
50% FBS, *t*_0_ = 1 min	55.13	0.96	0.03	32
75% FBS, *t*_0_ = 1 min	55.67	1.24	0.06	20.7
0% FBS (DI water), *t*_0_ = 1 min	72.36	0.41	0.05	8.2
0% FBS (DI water), *t*_0_ = 30 min	72.83	0.47	0.04	11.8
0% FBS (DI water), *t*_0_ = 120 min	72.69	0.46	0.07	6.6
5% FBS, *t*_0_ = 1 min	63.00	1.01	0.08	12.6
5% FBS, *t*_0_ = 30 min	55.46	0.87	0.09	9.7
5% FBS, *t*_0_ = 120 min	48.65	0.53	0.08	6.6
100% FBS, *t*_0_ = 1 min	52.91	1.90	0.06	31.7
100% FBS, *t*_0_ = 30 min	45.87	1.36	0.08	17
100% FBS, *t*_0_ = 120 min	44.84	0.54	0.12	4.5
100% FBS + SDS, *t*_0_ = 1 min	57.60	0.98	0.19	5.2
NP, *t*_0_ = 1 min	87.05	0.55	**0.25**	2.2
25% BSA, *t*_0_ = 1 min	46.10	0.77	0.05	15.4
Denatured 5% FBS, *t*_0_ = 1 min	70.37	0.94	0.21	4.5

### 2.2 Proof‐of‐Concept

**Figure 2:**
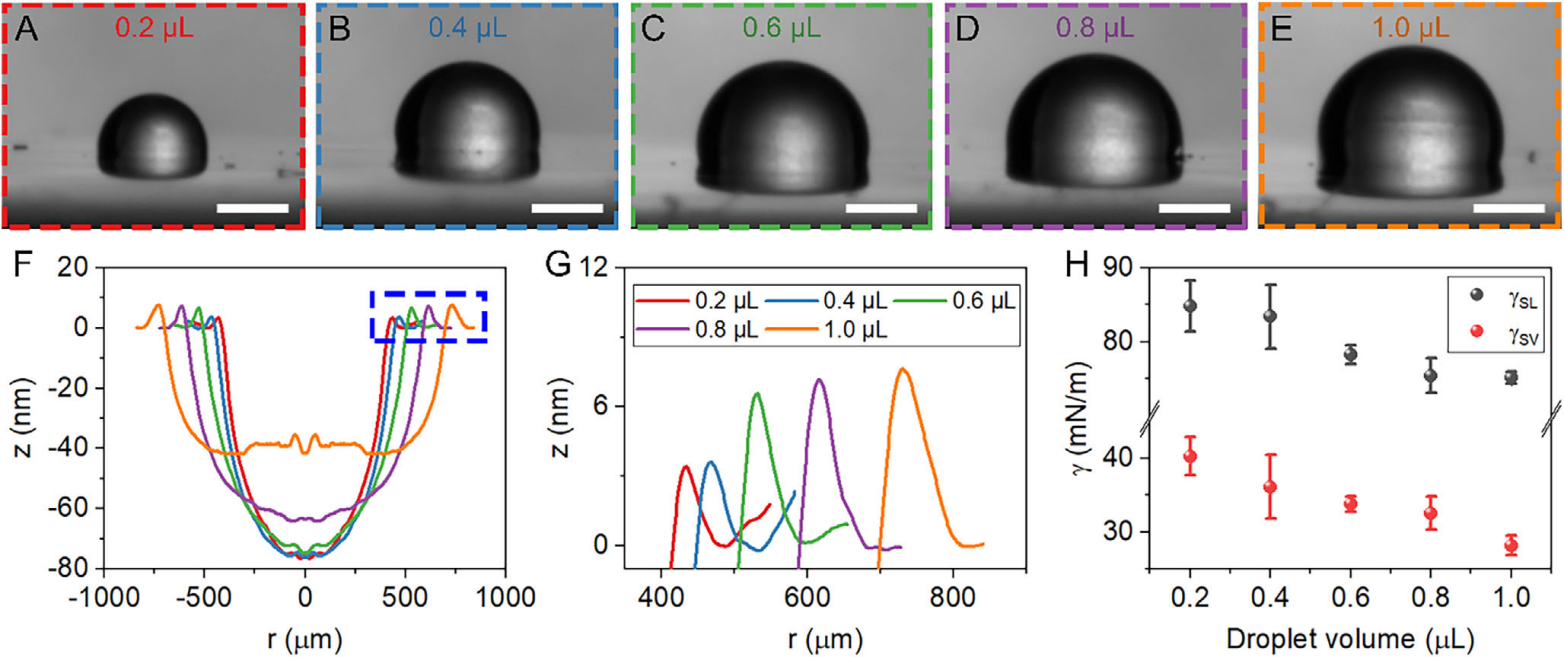
Deformation profiles and interfacial tensions of DI water drops. A–E) Sessile DI water drops of various volumes on the WiMTiP sensor. Scale: 0.5 mm. F) Azimuthal averaged deformation profiles. G) Wetting ridges of DI water drops of various volumes. H) Measurement of γ_*SL*_ and γ_*SV*_ with respect to the droplet volume (n =3).

Validation experiments are conducted with sessile DI water drops of various volumes over a WiMTiP sensor (**Figure** **2**A–E). All drops produce the characteristic substrate deformation that comprises a dimple immediately underneath the drop and a “lifting” wetting ridge at the three‐phase contact line (Figure 1E and Figure S3, Supporting Information). To better visualize the deformations and wetting ridge geometries, radial deformation profiles are obtained by transforming the 2D deformation map into a polar coordinate system and ensemble‐averaging in the azimuthal direction (Figure 2F). The wetting ridge height increases slowly but monotonically with drop volume, e.g., from 2.63 ± 0.84 nm for 0.2 µL to 6.54 ± 0.99 nm for 1.0 µL drops (Figure 2G). Our measurements agree well with the elastocapillary length (*l_E_*) of ≈10 nm^[^ ^37^ ^]^ for a water‐on‐PDMS system (*l*_E_ =  γ*E* ^−1^, where γ  =  20 *mN*/*m* ^[^ ^38^ ^]^ for DI water and *E* = 2 MPa for PDMS). Noticeable undulations are observed near the center, which are caused by fewer available data for averaging rather than by uncertainty in selecting the center points (Section S.5 and Figure S4, Supporting Information). Note that these fluctuations do not affect the wetting ridge geometry measurement. Additional trends, such as dimple depths and ridge heights with respect to drop volumes (Figure S5, Supporting Information), also agree well with the reported results.^[^ ^26^ ^]^ In short, the wetting ridge geometries are sufficiently resolved by the proposed technique.

To approximate instantaneous γ_*SL*_ and γ_*SV*_, the averaged radial profile is fitted at the ridge tip as two linear line segments intersected at the tip to obtain interface alignment, θ_2_ and θ_3_ (Figure 1D, Section S.6 and Figure S6, Supporting Information). With measurements of the water‐PDMS contact angle, θ_1_ = 109.6 ± 0.8° using a goniometer and the liquid‐vapor interfacial tension γ_*LV*_ = 72.8 ± 0.3 mN m^−1^ (Figure S7, Supporting Information), γ_*SL*_ and γ_*SV*_ are obtained by balancing γ_*SL*_, γ_*SV*_ and γ_*LV*_.^[^ ^26^ ^]^ Figure 2H shows the results of γ_*SL*_ and γ_*SV*_, whose values are consistent with literature, ranging from 16 to 185 mN m^−1[^ ^28^, ^30^, ^38^ ^]^ and 19 to 70 mN m^−1^,^[^ ^39^, ^40^ ^]^ respectively. Furthermore, a consistent decreasing trend is also observed for γ_*SL*_ and γ_*SV*_ that is attributed to the Shuttleworth effect, whereby a larger substrate deformation leads to a larger interfacial stress.^[^ ^39^ ^]^ Note that a larger drop produces a smaller deformation (Figure S5, Supporting Information).

### 2.3 Measuring Instantaneous γ_*SL*_ of Protein Mixtures

**Figure 3:**
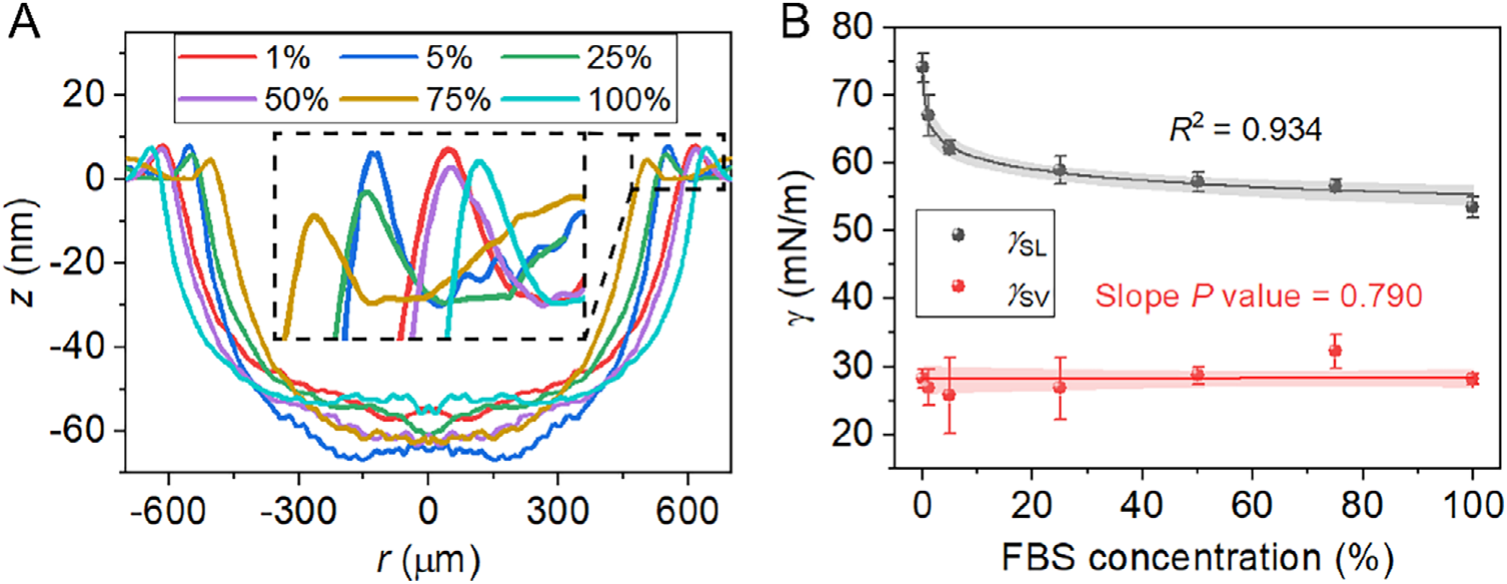
Deformation profiles and interfacial tensions of FBS drops. A) Deformation profiles of FBS drops with different concentrations. Inset: Close‐ups of the wetting ridges. B) γ_*SL*_ & γ_*SV*_ with respect to FBS concentrations (*n* = 3). Shaded areas represent the 95% confidence band of the fitted curves (nonlinear curve fitting).

A 0.4 µL drop of FBS, being a standard surrogate for blood fluid,^[^ ^41^ ^]^ is placed onto the sensor at different concentrations ranging from 1% to 100%. Note that a 0.4 µL drop is selected to ensure it fits in the FOV completely since FBS drops have a smaller contact angle and a larger base radius, while maintaining the accuracy in dispensing a small volume. As DI water drops, the deformation profiles of FBS drops show the same characteristics: dimples and wetting ridges (**Figures** **3**A and S8, Supporting Information). Measuring the contact angle, θ_1_, and γ_*LV*_ reveals a power‐law relationship with respect to the concentration, and this power‐law dependency is also observed in γ_*SL*_ (Figure 3B and Figure S9, Supporting Information) while γ_SV_ remains statistically constant (*P* value = 0.790, Figure 3B) since the water vapor‐PDMS interface is not modified by FBS concentration in suspensions. Collectively, these results, which are consistent with previous literature,^[^ ^34^, ^35^ ^]^ validate that the proposed sensor's ability to sufficiently resolve the instantaneous γ_*SL*_ for complex protein solutions and correlate the macroscopic γ_*SL*_ to molecular adsorption events of proteins.

### 2.4 Dynamics of γ_*SL*_

**Figure 4:**
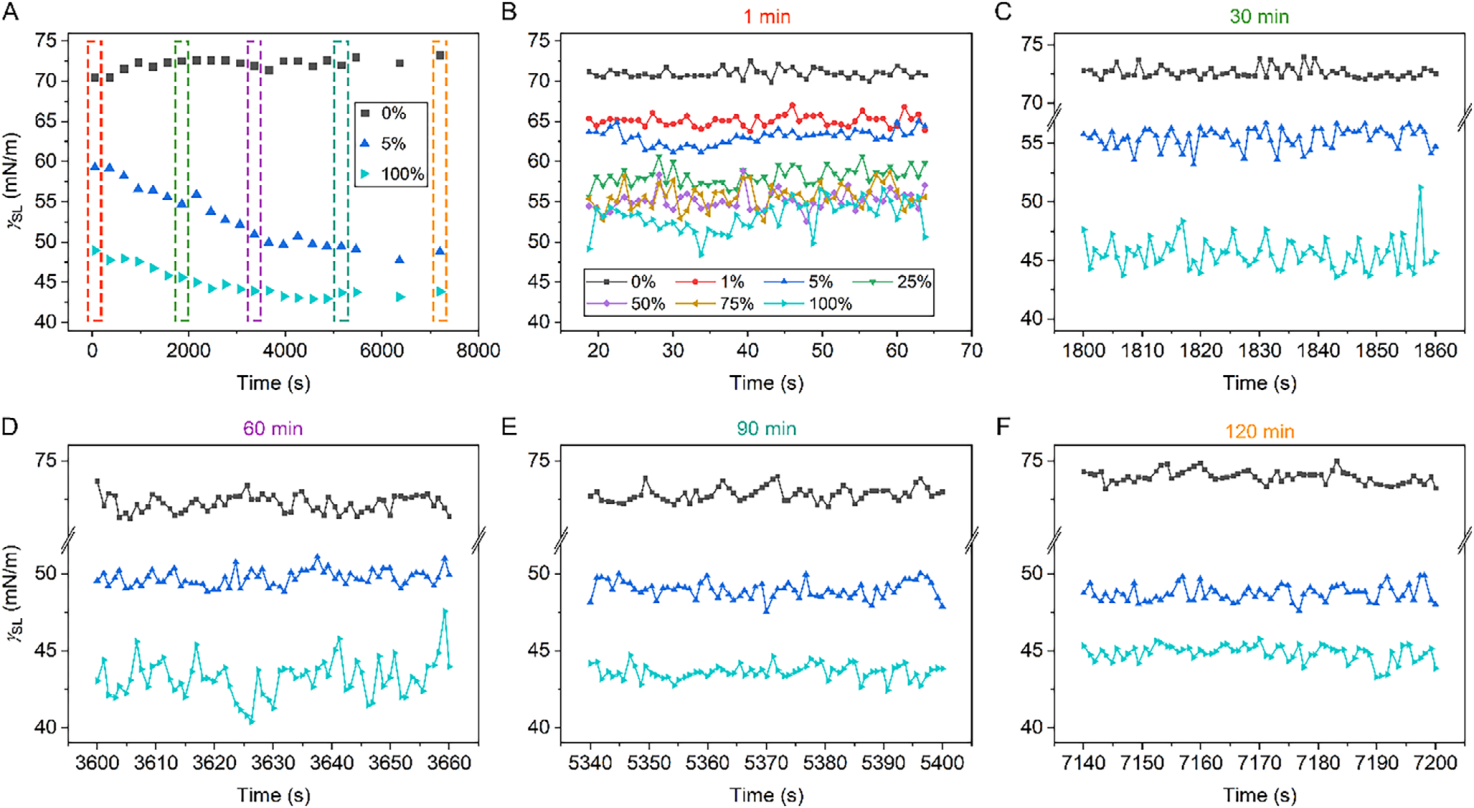
Temporal dynamics of the solid‐liquid surface tension. A) The coarse‐resolution dynamics of γ_*SL*_ sampled every 5 mins for 3 different FBS concentrations. B–E) The fine‐resolution dynamics of γ_*SL*_ sampled every 1 s at 5 different time windows as shown in (A).

The evolution of γ_*SL*_ is investigated by recording deformation profiles of 0.4 µL drops of various FBS concentrations for 2 h at an interval of ≈80 ms. Supplemented *a priori* by θ_1_ and γ_*LV*_ at various FBS concentrations over time (Figure S10A,B, Supporting Information), γ_*SL*_ at an interval of 5 mins is measured and shown in **Figure** **4**A. As expected, γ_*SL*_ of 0% FBS (i.e., DI water) remained unchanged throughout the 2 h, while γ_*SL*_ of 5% and 100% FBS showed a conventional Langmuir adsorption, i.e., an asymptotical decrease of γ_*SL*_ toward their equilibrium values (Table 1), while an increase of γ_*SV*_ to 30 mN m^−1^ irrespective of FBS concentration, showing temporal adsorption of water vapor over the substrate to equilibrium (Figure S10C, Supporting Information). Further scrutiny of γ_*SL*_ at an interval of 1 s reveals an intrinsic oscillation (Figure 4B–F). Due to prohibitive computation costs to analyze all recordings (e.g., one 2‐h dataset for one FBS concentration totalling 25,000 interferograms will take ≈3 years to be fully processed by a single I7 workstation), we only provide results of three selected concentrations (i.e., 0%, 5%, 100%) within five 1‐min windows starting at *t*_0_ =  1,  30,  60,  90,  120 min immediately after the drop deposition. We observe that oscillation amplitudes increase with FBS concentration (visually in Figure 4B and quantitatively in Table 1) and decrease with time (Figure 4C–F and Column 2 in Table 1) except for the control (i.e., 0% FBS). Since these oscillation amplitudes ($\sigma_{\gamma_{SL}^{\prime }}$, Column 2 in Table 1) are significantly larger than that of measurement uncertainty ($E_{\gamma_{SL}}$, Column 3 in Table 1), we conclude that the oscillations are real interfacial phenomena, not measurement noises. To further characterize these oscillations, Hilbert‐Huang transformation (HHT^[^ ^42^ ^]^) is applied to decompose the non‐stationary and intermittent γ_*SL*_(*t*) into $\gamma_{SL}^{R}+\sum \gamma_{SL,i}^{\prime }$ where $\gamma_{SL}^{R}$ is the low‐frequency undulating residual function and $\gamma_{SL,i}^{\prime }$ is the i‐th intrinsic model function (IMF) representing an oscillation at a specific frequency (Section S.8 and Figure S11A–G, Supporting Information). With these IMFs, we compute the characteristic total energy, *EG*, and expected mean frequency, *f*, to quantitatively characterize the γ_*SL*_ oscillations (Equation S8 and S9, Supporting information). In brief, the total energy of the oscillation is calculated as the sum of all the instantaneous energies in all IMFs obtained from the Hilbert‐Huang transformation (HHT), to quantify the oscillation amplitude. The frequency is calculated as the weighted average of the mean frequency of each IMF (details of the calculation can be found in Section S.8, Supporting Information).

**Figure 5:**
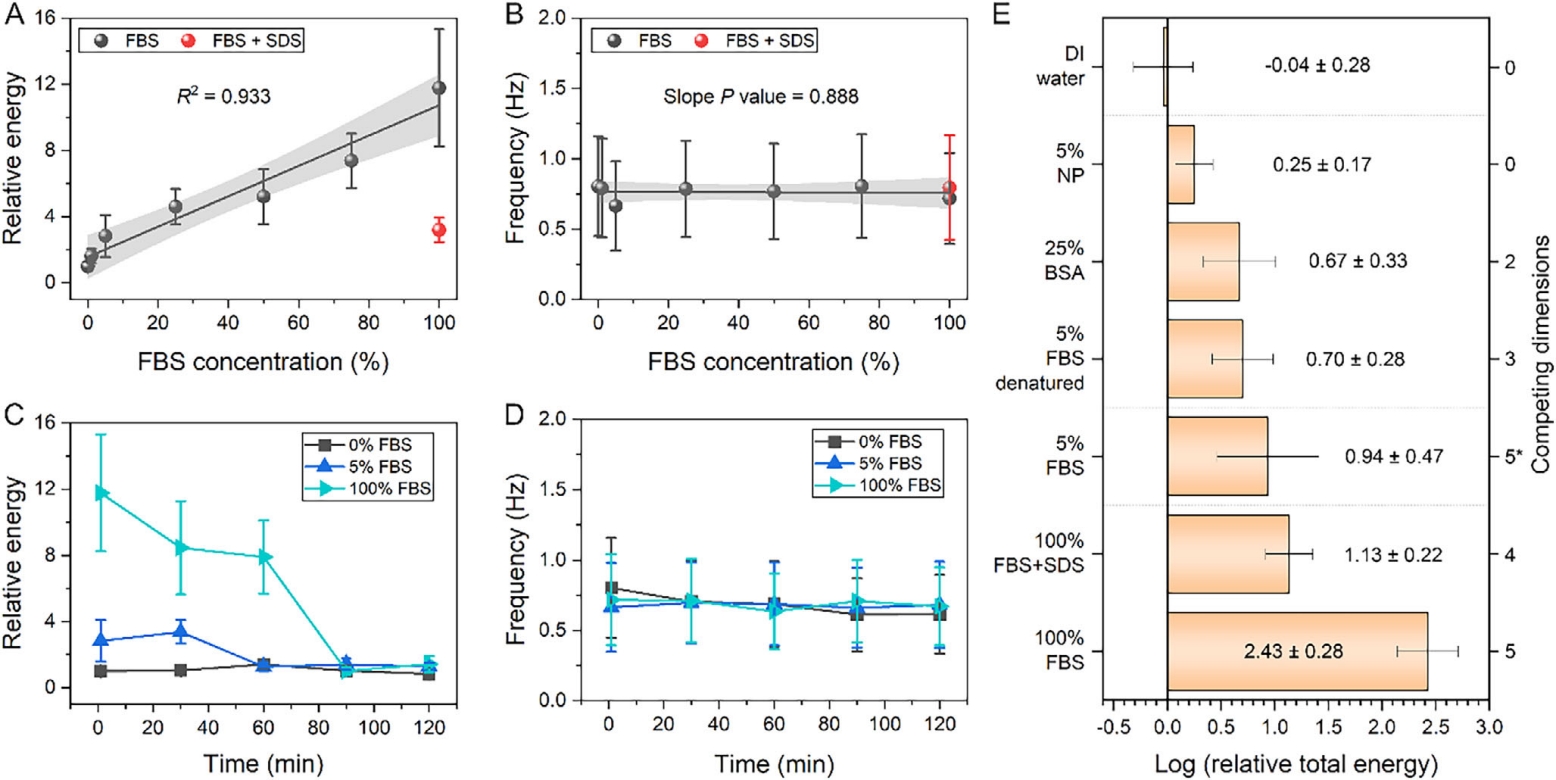
Quantification of the surface tension oscillation. A) The relative oscillation energy with respect to DI water, and B) oscillation frequency for different FBS concentrations and with surfactant SDS. Shaded areas represent the confidence band of the fitted curves. The temporal variation of the C) relative oscillation energy and D) mean oscillation frequency for 3 FBS concentrations sampled for 1 min intervals at 5 different time windows. E) The log relative oscillation energy at the first minute for different solutions. The asterisk highlights the case whereby the competing dimension is the same (e.g., same solution), the energy increases with the concentration (e.g., 5% FBS vs 100% FBS). Error bars: standard deviation obtained from 1000 bootstrap iterations.

Applying HHT analysis to γ_*SL*_(*t*) within a 1‐min window (Figure 4B) immediately after drop deposition (i.e. *t*_0_ =  1 min), we have found that the relative total energy (*RE* *G*_*FBS*/*DI*_ =  *EG_FBS_*/*EG_DI_*, where the subscript denotes the solution type) increases linearly with respect to concentration (**Figure** **5**A), while the *f* remains statistically constant (Figure 5B), quantitatively supporting our anecdotal observations in Figure 4B–F and Table 1. To elucidate the mechanism, we further conduct an experiment using a 100% FBS solution with 1% (w/v) sodium dodecyl sulfate (SDS) surfactant (Figure S11H, Supporting Information). We found that *REG*_*FBS* + *SDS*/*DI*_ is 3.7 times smaller than that of the 100% FBS solution (red dot in Figure 5A), as the surfactant blocks the protein adsorption. Compounded with results using solutions with different FBS concentrations, we confirm that the oscillation amplitude of γ_*SL*_is directly modulated by protein adsorption.

Applying HHT analysis to γ_*SL*_ measurements over 2 h (Figure 4A–F), we elucidate the time evolution of the oscillation characteristics (Figure 5 *C* and Figure S12, Supporting Information). The *REG*s for 5% and 100% FBS concentrations decrease over time as adsorption approaches equilibrium, while *REG_DI_* remains constant. Note that the decay time increases with the protein concentration (e.g., 60 min for 5% and 90 min for 100% FBS solution). Again, the *f* remains constant over time (Figure 5D).

To further examine the role of competitive adsorption in γ_*SL*_ kinematics, we have performed additional adsorption experiments using polystyrene NP, BSA, and denatured FBS drops and quantified *REG* at *t*_0_ =  1 min (natural logarithm of *REG* in Figure 5E). For the NP solution (presumably no competitive adsorption), the log of *REG* is only at 0.25 ± 0.17, which is significantly smaller than that of 100% FBS (e.g., 2.43 ± 0.28) but still statistically larger than that of DI water (e.g., 0 ± 0.28). It showcases that this oscillation is intrinsic to colloidal systems. When comparing single‐species BSA and multi‐species FBS, the log of *REG* for BSA is 3.6 times (or 1 order of magnitude) smaller. BSA is chosen because it is the main constituent of FBS,^[^ ^8^ ^]^ and the concentration is selected to be 25% (w/v), such that it contains 200 mg mL^−1^ (or 3 mM) of BSA protein in comparison to 25 mg mL^−1^ (or 0.4 mM) BSA protein found in 100% FBS.^[^ ^43^ ^]^ This disparity showcases that the oscillation amplitude is not solely dependent on concentration, but is also strongly affected by the presence of multiple protein species that compete for surface adsorption through size, conformation, and adsorption affinity, etc. Denaturing FBS by heat only led to a slight decrease in *REG*, since the majority of the above competing elements remain except for conformation. For brevity, we introduce the term, competing dimension, to represent the number of competing elements in the system hereinafter.

## 3 Discussions

The proposed sensor is unique in studying protein adsorption onto soft substrates by directly measuring the γ_*SL*_ in response to the molecular adsorption events and enabling their dynamic quantification, where the Young‐Dupre equation fails.^[^ ^36^ ^]^ We substantiate the claim by establishing the correlation of γ_*SL*_ to the protein concentrations (Figure 3) and resolving a typical Langmuir adsorption curve at a coarse temporal resolution (Figure 4A). Uncertainty analysis reveals that the measurement can achieve unprecedented accuracy of 0.25 mN m^−1^ (Table 1 and Section S.9, Supporting Information).

Using the FBS solution and time‐resolved γ_*SL*_, we discover, for the first time, a high‐frequency, low‐amplitude oscillation undulating the Langmuir curve. The oscillation amplitude increases with FBS concentrations (Figure 5A), which can be accounted for by a greater number of proteins undergoing adsorption at higher concentrations, and it decreases over time (Figure 5C), whereby the fraction of proteins undergoing irreversible adsorption increases with time.^[^ ^18^, ^44^, ^45^ ^]^ These observations, compounded with the facts of larger oscillation amplitudes in colloidal suspensions than those of DI water (control) and measured oscillation substantially exceeding measurement uncertainty, as well as our careful elimination of potential sources of temporal fluctuations during the measurements (e.g., γ_*LV*_ and contact angle in Figure S10, Supporting Information), provide clear evidence that the γ_*SL*_ oscillation is a manifestation of the adsorption/desorption events. To further solidify this notion, experiments using SDS showcase that the oscillation amplitude reduces significantly since SDS coating inhibits protein‐surface adsorption.^[^ ^46^ ^]^ Since this oscillation is also observed in simple NP, single‐species BSA, and denatured multi‐species FBS solutions, we conclude that it is intrinsic to any colloidal system

While the oscillation is an intrinsic property, its amplitude is greatly enhanced by the presence of competitive adsorption that is strongly affected by available competing elements such as protein sizes, affinity, conformations, number of protein species, and substrate properties (Figure 5E and Table 1). In general, any protein solution having most of the aforementioned elements causes the larger oscillations (Table S1, Supporting Information). Monodispersed NP solution, presumably having zero competing dimension, oscillates the least among all colloids and is slightly larger than that of the control. Unlike NP, BSA can undergo conformational change during adsorption that alters the affinity dynamically,^[^ ^47^ ^]^ and at high concentration, BSA aggregates, which leads to higher affinity.^[^ ^48^ ^]^ This leads to competitive adsorption in terms of size and affinity, which explains the 2.7‐fold increase in the log of *REG* compared to NP. However, this situation constitutes a relatively simple case of competitive adsorption of two colloids, i.e., single and aggregated BSA. When scrutinizing the case of FBS, which is known to undergo competitive adsorption,^[^ ^8^, ^22^, ^49^, ^50^, ^51^ ^]^ this represents a much more complex scenario with hundreds of protein species with different affinities, sizes, and conformations.^[^ ^50^ ^]^ The larger the competing dimension, the larger the oscillation. Denaturing the proteins led to permanent conformation changes that fixed the protein affinity,^[^ ^52^ ^]^ reducing competing dimension, which explains only a slight reduction (1.3 times) in the oscillation amplitude for the denatured FBS in comparison to its unmodified counterparts. Note that the γ_*SL*_ uncertainty for the DI water is ≈5 times smaller than the fluctuations amplitude (Column 3 in Table 1) and this supports our assertion that the sensor is capturing the dynamic wetting/dewetting by water molecules at the solid surface, in which prior literature has reported the broken symmetry of water molecules as a mechanism of inducing preferential orientations, and thus, competition, toward the solid surface.^[^ ^53^ ^]^ Further investigation on the subject is needed. Note that while the origins of γ_*SL*_ oscillation may be attributed to individual competing elements, such as protein type, size, conformation, affinity, electric double‐layer, and solution pH, ionic strength, hydration gradient, and equilibration from the solid phase, our results quantitatively correlate the increase of oscillation amplitudes with the increase of competitive dimensions (Figure 5E). Future research on how each competing element (above) affects the γ_*SL*_ oscillation is warranted.

One alternative hypothesis to the competitive adsorption model is the conventional concentration narrative, i.e., when the protein concentration increases, more adsorption and desorption events occur, leading to a greater oscillation amplitude. This is true for solutions with the same chemical composition but with different concentrations (Figure 5A). However, contrary to the concentration model prediction, our results (Figure 5E) show that the γ_*SL*_ oscillation amplitude by 25% (w/v) BSA (or 3 mM) at 0.67 ± 0.33 is much smaller than that by 100% (v/v) FBS (or 0.4 − 0.6 mM) at 2.43 ± 0.28. Since the major constituents of FBS (e.g., the mean molecular mass of 70.5 kDa) are BSA (e.g., 66.5 kDa), the contribution of surface affinity to oscillation is expected to be similar between the two suspensions. However, the above unexpected result, opposite to the concentration model prediction, nullifies the concentration model as an alternative interpretation. The competitive adsorption model that relates the competitive dimensions of the solutions to the oscillation amplitude remains the one model that coherently explains all results in this study.

It is well known that using a shorter‐wavelength (e.g., blue or UV) laser will increase the measurement sensitivity of a DHI,^[^ ^54^ ^]^ but it can also lead to greater damage (e.g., aggregation, denaturation, and ionization) to biological specimens, including proteins,^[^ ^55^, ^56^ ^]^ cells, tissues, and microorganisms. Care must be taken to balance the benefits to improve measurement sensitivity and the damage to the specimen when one selects the DHI laser. One obvious limitation of the proposed interferometric nanostrain sensor technology is the computational cost required to obtain the deformation field. For instance, a single 2K × 2K DHI recording will require ≈1 h to process using a 12‐core I7 workstation. Recent development has shown that this processing bottleneck can be circumvented by employing deep learning or artificial intelligence to significantly boost the processing efficiency by 4500‐fold, which completes a 2 h measurement within 6 h. This development will be reported in future communications.

## 4 Conclusion

A soft interferometric nanostrain sensor is introduced to study protein adsorption by directly measuring instantaneous γ_*SL*_ via the elastocapillary effect. Using this sensor, a high‐frequency, low‐amplitude oscillation of γ_*SL*_ is observed to be superimposed on top of a typical Langmuir adsorption, which is revealed to be an intrinsic property of colloidal systems adsorbing onto solid substrates. Quantification of this oscillation revealed a dependency of the oscillation energy on the concentration and time. The presence of competitive adsorption will further amplify the oscillation amplitude. This interferometric nanostrain sensor, while adopting the ensemble averaging approach, provides a potential method to probe interfacial protein phenomena with a high spatiotemporal resolution and subsequently enables the direct observation as well as provides a means to quantify the molecular competitive protein adsorption via instantaneous γ_*SL*_, which will be crucial in validating molecular dynamic simulations of competitive adsorption.^[^ ^57^ ^]^

## 5 Experimental Section

The experimental section is briefly summarized below and detailed in the Supporting Information.

### Fabrication of the WiMTiP Sensor

The fabrication of the WiMTiP sensor has been reported by our group.^[^ ^27^ ^]^ Briefly, a layer of PDMS (monomer: crosslinker ratio = 10:1) was cast and cured on a glass substrate. A jammer (e.g., parylene‐C) of thickness ≈160 nm was then deposited by physical vapor deposition (Parylene 2010 Labcoater, Specialty Coating Systems Inc.). A 50 nm aluminum film was sputtered (ATC 2000, AJA International Inc.). A 20 µm PDMS layer was then spin‐coated over the nm Al film.

### Data Processing

The processing methods were originally developed by Zhang et al.^[^ ^58^ ^]^ and our improved implementation is described in Section S.2 (Supporting Information).

### Solution Preparation and Deposition

FBS solution (pH = 7.3, ionic strength = 0.10–0.15 m,^[^ ^59^, ^60^, ^61^ ^]^ 97068–085, VWR) was diluted using DI water to the desired concentration (v/v). Note that multiple lots of FBS from the same supplier have been used for the experiments. Typical components found in FBS are shown in Table S2 (Supporting Information). The average molar concentration of FBS was calculated using a typical total protein concentration of 30 – 45 mg mL^−1[^ ^62^ ^]^ and the average molecular mass of the proteins listed in Table S2 (Supporting Information). The datasheet for the supplier's FBS can be found at.^[^ ^63^ ^]^ BSA solution (pH = 7.5, BP1600‐100, Fisher Scientific) was prepared by dissolving the lyophilized heat shock‐treated BSA powder in DI water (w/v). Similarly, the NP solution was prepared by dispersing 200 nm polystyrene nanoparticles (1% solids, pH = 7.8, diameter coefficient of variation (CV) <2%, density = 1.05 g cm^−3^, 3200A, ThermoFisher) in DI water. The pH value of the solution was measured using a pH meter (MW 101, Milwaukee). The denaturing of FBS was achieved by heating the FBS solution in a water bath at 100 °C for 1 h.^[^ ^64^ ^]^ After cooling, additional DI water was added to ensure the concentration was the same as before denaturation. All solutions were deposited onto the WiMTiP sensor surface using a syringe pump (NE‐1000, New Era Pump System Inc.) via a Tygon tubing with a micropipette tip. The deposition volume was fixed at 0.4 µL. A small plastic box was placed over the droplets and sealed with parafilm to minimize evaporation during experiments. No volume change was observed during 2 h experiments.

### Contact Angle and γ_*LV*_ Measurements

Images of sessile DI water, FBS, and BSA solution deposited on the WiMTiP sensor surface, as well as pendant drops, were captured using an in‐house goniometer. The contact angles and liquid‐vapor interfacial tension were extracted using the ImageJ plugin ‘DropSnake’^[^ ^65^ ^]^ and ‘Pendent_Drop’,^[^ ^66^ ^]^ respectively.

### Hilbert‐Huang Transformation

The γ_SL_ was first decomposed into IMFs with specific oscillation frequencies and residuals. The IMFs were analyzed by Hilbert transformation to obtain power spectra and total energy of each intrinsic mode, *a.k.a*. Hilbert‐Huang transformation.^[^ ^67^ ^]^ The total energy of oscillation modes (i.e., without residual) was summed together. The mean oscillation frequency was conditionally averaged over all oscillation modes (Section S.8, Equation S8 and S9, Supporting Information) to quantify the overall oscillation frequency of γ_*SL*_.

### Statistical Analysis

Values were represented as the mean ± SD. Curve fitting was done with each data point assigned a weight *w_i_* = Δ*y* ^−2^ , where Δ*y* denotes the error. The adjusted *R* ^2^ value was reported if the fitted curve was significantly different from *y*  =  0, whereas the *P* value was reported if the fitted curve was not significantly different from *y*  =  0. The error for the γ_SL_ time series data was estimated using stationary bootstrapping^[^ ^68^ ^]^ with 1000 iterations, and the optimal block length for the stationary bootstrapping was estimated using the methodology proposed by Politis and White.^[^ ^69^ ^]^ A *P* value < 0.05 was considered statistically significant. All statistical analysis was done using MATLAB 2021b (MathWorks Inc.) and Origin Pro 2021 (OriginLab Inc.).

## Conflict of Interest

The authors declare no conflict of interest.

## Supporting information



Supporting Information

## Data Availability

The data that support the findings of this study are available from the corresponding author upon reasonable request.

## References

[1] Y. Engel , J. D. Schiffman , J. M. Goddard , V. M. Rotello , Mater. Today 2012, 15, 478.

[2] M. Hedayati , M. J. Neufeld , M. M. Reynolds , M. J. Kipper , Materials Science and Engineering: R: Reports 2019, 138, 118.

[3] E. A. Vogler , Biomaterials 2012, 33, 1201. 22088888 10.1016/j.biomaterials.2011.10.059 PMC3278642

[4] P. Roach , D. Farrar , C. C. Perry , J. Am. Chem. Soc. 2005, 127, 8168. 15926845 10.1021/ja042898o

[5] C. F. Wertz , M. M. Santore , Langmuir 2002, 18, 1190.

[6] M. Rabe , D. Verdes , S. Seeger , Adv. Colloid Interface Sci. 2011, 162, 87. 21295764 10.1016/j.cis.2010.12.007

[7] H. P. Felgueiras , N. S. Murthy , S. D. Sommerfeld , M. M. Brás , V. Migonney , J. Kohn , ACS Appl. Mater. Interfaces 2016, 8, 13207. 27144779 10.1021/acsami.5b12600 PMC6707081

[8] R.‐Q. Hou , N. Scharnagl , R. Willumeit‐Römer , F. Feyerabend , Acta Biomater. 2019, 98, 256. 30771533 10.1016/j.actbio.2019.02.013

[9] S. Angioletti‐Uberti , M. Ballauff , J. Dzubiella , Mol. Phys. 2018, 116, 3154.

[10] I. Lynch , A. Salvati , K. A. Dawson , Nat. Nanotechnol. 2009, 4, 546. 19734922 10.1038/nnano.2009.248

[11] C. J. Wilson , R. E. Clegg , D. I. Leavesley , M. J. Pearcy , Tissue Eng. 2005, 11, 1. 15738657 10.1089/ten.2005.11.1

[12] M. A. Butt , Biosensors 2025, 15, 35. 39852086 10.3390/bios15010035 PMC11763797

[13] D. Johannsmann , I. Reviakine , Nature Reviews Methods Primers 2024, 4, 63.

[14] L. Ma , D. Pang , C. Deng , Colloids Surf. Physicochem. Eng. Aspects 2019, 582, 123860.

[15] M. Tagaya , T. Ikoma , N. Hanagata , T. Yoshioka , J. Tanaka , Sci. Technol. Adv. Mater. 2011, 12, 034411. 27877402 10.1088/1468-6996/12/3/034411 PMC5090474

[16] R. J. Green , M. C. Davies , C. J. Roberts , S. J. B. Tendler , Biomaterials 1999, 20, 385. 10048412 10.1016/s0142-9612(98)00201-4

[17] M. Hedayati , D. F. Marruecos , D. Krapf , J. L. Kaar , M. J. Kipper , Acta Biomater. 2020, 102, 169. 31731023 10.1016/j.actbio.2019.11.019

[18] M. Hedayati , D. Krapf , M. J. Kipper , J. Colloid Interface Sci. 2021, 589, 356. 33482534 10.1016/j.jcis.2021.01.001

[19] X. Chen , Y. Wang , X. Zhang , C. Liu , Biomater. Sci. 2021, 9, 5484. 34286716 10.1039/d1bm00676b

[20] C. Manzo , M. F. Garcia‐Parajo , Rep. Prog. Phys. 2015, 78, 124601. 26511974 10.1088/0034-4885/78/12/124601

[21] M. Malmsten , B. Lassen , J. Colloid Interface Sci. 1994, 166, 490.

[22] B. Lassen , M. Malmsten , J. Colloid Interface Sci. 1997, 186, 9. 9056289 10.1006/jcis.1996.4529

[23] S. J. Kapp , I. Larsson , M. Van De Weert , M. Cárdenas , L. Jorgensen , J. Pharm. Sci. 2015, 104, 593. 25446557 10.1002/jps.24265

[24] L. Yin , W. Wang , S. Wang , F. Zhang , S. Zhang , N. Tao , Biosens. Bioelectron. 2015, 66, 412. 25486538 10.1016/j.bios.2014.11.036 PMC4836836

[25] L. Hauer , A. Naga , R. G. M. Badr , J. T. Pham , W. S. Y. Wong , D. Vollmer , Soft Matter 2024, 20, 5273. 38952198 10.1039/d4sm00346b

[26] R. W. Style , R. Boltyanskiy , Y. Che , J. S. Wettlaufer , L. A. Wilen , E. R. Dufresne , Phys. Rev. Lett. 2013, 110, 066103. 23432280 10.1103/PhysRevLett.110.066103

[27] M. Jalali‐Mousavi , S. K. S. Cheng , J. Sheng , Nanomaterials 2023, 13, 1044. 36985941 10.3390/nano13061044 PMC10054355

[28] S. Mondal , M. Phukan , A. Ghatak , Proc. Natl. Acad. Sci. U.S.A. 2015, 112, 12563. 26420871 10.1073/pnas.1502642112 PMC4611640

[29] N. Singh , A. Kumar , A. Ghatak , J. Colloid Interface Sci. 2023, 645, 266. 37150000 10.1016/j.jcis.2023.04.103

[30] N. Nadermann , C. Y. Hui , A. Jagota , Proc. Natl. Acad. Sci. U.S.A. 2013, 110, 10541. 23754440 10.1073/pnas.1304587110 PMC3696820

[31] E. R. Jerison , Y. Xu , L. A. Wilen , E. R. Dufresne , Phys. Rev. Lett. 2011, 106, 186103. 21635105 10.1103/PhysRevLett.106.186103

[32] Y. S. Chu , J. M. Yi , F. De Carlo , Q. Shen , W.‐K. Lee , H. J. Wu , C. L. Wang , J. Y. Wang , C. J. Liu , C. H. Wang , S. R. Wu , C. C. Chien , Y. Hwu , A. Tkachuk , W. Yun , M. Feser , K. S. Liang , C. S. Yang , J. H. Je , G. Margaritondo , Appl. Phys. Lett. 2008, 92, 103119.

[33] S. J. Park , B. M. Weon , J. S. Lee , J. Lee , J. Kim , J. H. Je , Nat. Commun. 2014, 5, 4369. 25007777 10.1038/ncomms5369 PMC4104447

[34] W. van der Vegt , W. Norde , H. C. van der Mei , H. J. Busscher , J. Colloid Interface Sci. 1996, 179, 57.

[35] J. Noordmans , H. Wormeester , H. J. Busscher , Colloids Surf., B 1999, 15, 227.

[36] R. W. Style , A. Jagota , C.‐Y. Hui , E. R. Dufresne , Annu. Rev. Condens. Matter Phys. 2017, 8, 99.

[37] J. Bico , É. Reyssat , B. Roman , Annu. Rev. Fluid Mech. 2018, 50, 629.

[38] M. J. Owen , Macromol. Rapid Commun. 2021, 42, 2000360. 10.1002/marc.202000360 32935908

[39] Q. Xu , K. E. Jensen , R. Boltyanskiy , R. Sarfati , R. W. Style , E. R. Dufresne , Nat. Commun. 2017, 8, 555. 28916752 10.1038/s41467-017-00636-y PMC5601460

[40] X. Xu , A. Jagota , D. Paretkar , C.‐Y. Hui , Soft Matter 2016, 12, 5121. 27189735 10.1039/c6sm00584e

[41] A. J. Galante , S. Haghanifar , E. G. Romanowski , R. M. Q. Shanks , P. W. Leu , ACS Appl. Mater. Interfaces 2020, 12, 22120. 32320200 10.1021/acsami.9b23058

[42] M.‐T. Lo , V. Novak , C. K. Peng , Y. Liu , K. Hu , Phys. Rev. E 2009, 79, 061924. 10.1103/PhysRevE.79.061924 PMC2730740 19658541

[43] K. T. Huang , Y. H. Chen , A. M. Walker , BioTechniques 2004, 37, 406. 15470895 10.2144/04373ST05

[44] C. Calonder , Y. Tie , P. R. Van Tassel , Proc. Natl. Acad. Sci. U.S.A. 2001, 98, 10664. 11535805 10.1073/pnas.181337298 PMC58523

[45] A. Abdelrasoul , N. Zhu , H. Doan , A. Shoker , Sci. Rep. 2023, 13, 1692. 36717597 10.1038/s41598-023-27596-2 PMC9886930

[46] G. T. Roman , S. Carroll , K. McDaniel , C. T. Culbertson , Electrophoresis 2006, 27, 2933. 16721904 10.1002/elps.200500795

[47] M.‐H. Seo , J. Park , E. Kim , S. Hohng , H.‐S. Kim , Nat. Commun. 2014, 5, 3724. 24758940 10.1038/ncomms4724

[48] X. Cao , J. Li , X. Yang , Y. Duan , Y. Liu , C. Wang , Thermochim. Acta 2008, 467, 99.

[49] Y. Arima , H. Iwata , Acta Biomater. 2015, 26, 72. 26306676 10.1016/j.actbio.2015.08.033

[50] J. Deng , T. Ren , J. Zhu , Z. Mao , C. Gao , Regenerative Biomaterials 2014, 1, 17. 26814446 10.1093/rb/rbu008 PMC4669003

[51] J. X. Xu , N. C. Fitzkee , Front. Physiol. 2021, 12, 2021. 10.3389/fphys.2021.715419 PMC8415878 34483968

[52] J. H. Park , J. A. Jackman , A. R. Ferhan , G. J. Ma , B. K. Yoon , N.‐J. Cho , ACS Appl. Mater. Interfaces 2018, 10, 32047. 30178663 10.1021/acsami.8b13749

[53] R. Walker‐Gibbons , A. Kubincová , P. H. Hünenberger , M. Krishnan , J. Phys. Chem. B 2022, 126, 4697. 35726865 10.1021/acs.jpcb.2c01752 PMC9251758

[54] S. Schedin , G. Pedrini , H. J. Tiziani , A. K. Aggarwal , M. E. Gusev , Appl. Opt. 2001, 40, 100. 18356978 10.1364/ao.40.000100

[55] E. I. Nagaev , I. V. Baimler , A. S. Baryshev , M. E. Astashev , S. V. Gudkov , Molecules 2022, 27, 6752. 36235285 10.3390/molecules27196752 PMC9573762

[56] A. A. C. Wainwright , K. Madhoun , P. Su , S. E. Janisse , J. E. Besaw , H. S. Grewal , O. P. Ernst , J. O. Kafader , N. L. Kelleher , R. J. D. Miller , J. Phys. Chem. Lett. 2025, 16, 8785. 40833879 10.1021/acs.jpclett.5c01440 PMC12969999

[57] L. A. Pugnaloni , E. Dickinson , R. Ettelaie , A. R. Mackie , P. J. Wilde , Adv. Colloid Interface Sci. 2004, 107, 27. 14962406 10.1016/j.cis.2003.08.003

[58] C. Zhang , R. Miorini , J. Katz , Exp. Fluids 2015, 56, 203.

[59] M. Efstratiou , J. Christy , D. Bonn , K. Sefiane , Colloids and Interfaces 2021, 5, 43.

[60] M. Efstratiou , J. R. E. Christy , D. Bonn , K. Sefiane , Langmuir 2022, 38, 4321. 35357835 10.1021/acs.langmuir.2c00019 PMC9009182

[61] J. Yu , L. Zhang , IEEE/ASME Transactions on Mechatronics 2019, 24, 154.

[62] A. R. Kolli , Clin. Transl. Sci. 2023, 16, 2123. 37605430 10.1111/cts.13616 PMC10651662

[63] https://www.avantorsciences.com/us/en/product/18706422/avantor‐seradigm‐premium‐grade‐fetal‐bovine‐serum‐fbs?isCatNumSearch=true.

[64] S. Kumar , E. Lazau , C. Kim , Int J Nanomedicine 2021, 16, 3707. 34103912 10.2147/IJN.S307027 PMC8180297

[65] A. F. Stalder , T. Melchior , M. Müller , D. Sage , T. Blu , M. Unser , Colloids Surf. Physicochem. Eng. Aspects 2010, 364, 72.

[66] A. Daerr , A. Mogne , Journal of Open Research Software 2016, 4.

[67] N. E. Huang , Hilbert‐Huang transform and its applications, World Scientific 2014, 16.

[68] D. N. Politis , J. P. Romano , J. Am. Stat. Assoc. 1994, 89, 1303.

[69] D. N. Politis , H. White , Econometric Reviews 2004, 23, 53.

