# Supplementary material for: Soft Interferometric Nanostrain Sensor Reveals Solid‐Liquid Interfacial Tension Oscillation Amplified by Competitive Adsorption
Source: Small. 2025 Dec 8;22(6):e08858. doi: 10.1002/smll.202508858 (PMC12837351; doi:10.1002/smll.202508858)
Supplement: Supplementary file 1 — Supporting Information [file SMLL-22-e08858-s001.pdf]

NANO · MICRO
small

Supporting Information

for *Small*, DOI 10.1002/smll.202508858

Soft Interferometric Nanostrain Sensor Reveals Solid-Liquid Interfacial Tension Oscillation Amplified by Competitive Adsorption

*Samuel K.S. Cheng, Maryam Jalali-Mousavi and Jian Sheng**

Supporting Information

Soft Interferometric Nanostrain Sensor Reveals Solid-Liquid Interfacial Tension Oscillation Amplified by Competitive Adsorption

*Samuel K. S. Cheng, Maryam Jalali-Mousavi, Jian Sheng**

College of Engineering and Computer Science, Texas A&M University-Corpus Christi, Corpus Christi, TX 78412, USA

*Corresponding author
Email: jian.sheng@tamucc.edu

Expanded Experimental Section

S.1 Optical Setup of Digital Holographic Interferrometry (DHI). The main components of DHI (Fig. 1A) include a 25 mW, 633 nm He-Ne laser (R-30995, Newport), a pin-hole assembly comprised of a 10X microscope objective, 25-μm pin holes, an aspheric lens (AL50100M-A, Thorlabs), 4 mirrors (one BB1-E02 and three BB2-E02, Thorlabs), a beamsplitter (BS031, Thorlabs), a 50-mm right-angle prism (PS919, Thorlabs), a projection lens (AF Nikkor 105 mm, Nikon), and an infinite corrected microscope, where the last two components form an in-house ultra long variable working distance (ULVWD) microscope with the minimum working distance of 150 mm (bottom Fig. 1A). The in-house developed infinite-corrected microscope consists of an infinite corrected semi-plane objective (e.g. SP 10X, Motic), a tube lens (TTL200-S8, Thorlabs) with a tube length of 200 mm, and a charge-coupled device (CCD) camera (CLB-B2021M-TF000, Imperx). Note that the imaging plane of the projection lens is placed right at the resting place of the nm metallic thin film or flexible mirror (Fig. 1A). The back imaging plane of the projection lens is the front object plane of the microscope, whose back imaging plane (or imaging plane of the tube lens) overlaps with the camera's sensor. Further noticed that we use a Nikon Nikkor 105 zoom-in lens as our project lens to achieve variable working distance and magnifications with a superior spatial resolution (>1,000 lines/mm at f/2.8). The alignment of ULVWD is performed a priori and integrated into the rest of the system by aligning the pickup prism and matching the optical paths of both beams. It is also worth noting that the camera is connected to a swappable mount that allows us to interchange a low-speed speed large-format CCD camera with other cameras, including high-speed, large-format CMOS cameras (IDT N4, Innovative Imaging).

A brief description of the imaging principle is the following: a collimated laser beam enters the 50:50 beam splitter (BS) and is split into the object and reference beams. The objective beam illuminating the WiMTiP sensor (e.g., 50 nm metallic thin film) from below is then reflected by it, whereas the reference beam is directly reflected by a 2" flat mirror. The two beams are then combined by the same BS and redirected by the pickup prism towards the ULVWD microscope, and interferograms are recorded by the camera.

S.2 DHI Data Processing. The processing steps of the interferograms have been previously reported.[1] First, the min/max filter is applied to the raw interferograms, whereby each pixel intensity in the interferograms is normalized using 200 frames in the time sequence, followed by a 5 × 5 pixels spatial median filter. This step aims to compensate for the spatial nonuniform intensity variation in the interferograms. The next processing step involves auto/cross-

correlation filtering. The max/min filtered interferogram is split into $m \times n$ pixels window, and a mask containing 1 fringe cycle in the autocorrelation map of the window is extracted. This mask is then cross-correlated with the min/max filtered interferogram to produce a locally filtered image, and the center 5 × 5 pixel blocks in the noisy min/max filtered interferogram are replaced with the corresponding value in the locally filtered image. This process is repeated for all 5 × 5 pixel blocks in the interferogram to obtain the auto/cross-correlation filtered interferograms. The extraction of the phase ϕ is first performed by taking the arccosine of the interferogram and unwrapping the phase so that ϕ ranges from 0 to 2π. To correct any defects in the interferogram, the gradients of the interferogram, i.e., $\frac{d\phi}{dx}$ and $\frac{d\phi}{dy}$ are calculated, and any large discontinuities are detected using a threshold, removed, and interpolated to obtain a smooth gradient field. Last, the phase field is obtained by integrating the corrected gradient field.

To obtain the deformation field of the sessile droplets, the phase field of an undeformed frame is subtracted from the frame with the sessile droplet to correct for any preexisting background deformation. Next, the large feature in the corrected phase field, i.e., the center dimple, is cropped out before fitting a 5th-order polynomial to estimate the background bias. The cropping is necessary as large features will distort the estimation of the background bias. A 5th-order polynomial is chosen because lower-order corrections (i.e., first to third order) are not able to remove optical aberrations, and a 5th order has better results compared to a 4th order visually (Figure S13, Supporting Information). Note that the background correction is performed for every frame. Lastly, the background bias is subtracted from the corrected phase field and converted into the deformation field using the following formula:

$$\phi = \frac{2\pi}{\lambda}\eta_{PDMS}(2d) \tag{S1}$$

where λ = 633 nm being the wavelength of the laser, η_{PDMS} = 1.43 being the refractive index of PDMS, and d being the deformation.

S.3 *Z* Resolution estimation. Fifteen error maps, defined as the difference between the deformation profiles of two undeformed frames, are first calculated. The noise floor for each error map is calculated as follows:

$$noise\ floor = \sqrt{\frac{1}{n}\sum_{i=1}^{n} z_i^2} \quad (S2)$$

where n is the number of pixels and z is the z-direction deformation value. The resolution is defined as the average of the noise floor across all 15 error maps.

S.4 Nanoindentation experiments. For the elastic modulus characterization, nanoindentation is performed using an atomic force microscope (TT-2 AFM, AFM Workshop). The cantilever used has an elastic modulus of 5.3889 Pa and a pyramidal tip. Ten force-indentation curves are performed at randomly selected locations, and the curves are fitted with the standard linear solid (SLS) model with Ting's integral to obtain the elastic modulus at each location.[2] The final elastic modulus of the sensor is estimated by averaging all 10 locations.

S.5 Analysis of deformation map generated by a sessile drop. Starting with the 2D deformation profiles of the sessile droplets, a circle is first fitted using ImageJ to locate the center point. The line profile passing through the center point is then averaged over the azimuthal direction to obtain a radial deformation profile in the polar coordinates. The wetting ridge height is defined as the difference between the maximum and minimum of the wetting ridge. Given the observed undulation near the dimple, the extraction of the dimple height first involved normalizing the radial deformation profile with respect to the radius of the droplet R (i.e., distance from r = 0 to the maximum). Next, data within the range of -0.75 r/R to 0.75 r/R is used to fit a 2nd order polynomial curve, and the dimple height is taken as the z value at r/R = 0 of the fitted curves (Figure S5, Supporting Information).

S.6 Extraction of the Solid-liquid and Solid-vapor Interfacial Tension. The extraction of the solid-liquid and solid-vapor interfacial tension is performed using the radial deformation profile. Due to the azimuthal averaging and the limited $x - y$ spatial resolution, the measured wetting ridge tip will be inherently smoothed. Thus, to obtain θ_2 and θ_3, the wetting ridge tip (data where $z \geq 0$) is first split into a left and a right profile at the maximum point. One-third of each profile, starting from z = 0 to the maximum point, is then used to fit a linear line to extract θ_2 and θ_3 (Figure S6, Supporting Information). θ_1 is obtained from the contact angle measurement, and the liquid-vapor interfacial tension γ_{LV} is obtained from the pendant drop

measurements. With the above information, the solid-liquid γ_{SL} and solid-vapor γ_{SV} interfacial tension can then be solved using the following equations:

$$\gamma_{LV}\cos\theta_1 + \gamma_{SL}\cos\theta_2 = \gamma_{SV}\cos\theta_3 \tag{S3}$$

$$\gamma_{LV}\sin\theta_1 = \gamma_{SV}\sin\theta_3 + \gamma_{SL}\sin\theta_2 \tag{S4}$$

For the long-term adsorption experiments, the rloess (robust locally estimated scatterplot smoothing) regression with a span of 0.9 is used to obtain a smooth curve for the contact angles and γ_{LV} over the span of two hours. The γ_{SL} and γ_{SV} is then calculated using Equation S3 and S4 using the smoothed data. This is to remove any possible oscillations arising from the contact angle and γ_{LV} data.

S.7 Contact Angle and Liquid-vapor Interfacial Tension Measurement. Images of sessile DI water, FBS, and BSA solution are measured using an in-house goniometer, which consists of a projection lens (AF Nikkor 105 mm, Nikon) connected to a CCD camera (CLB-B2021M-TF000, Imperx) and a light source. The solutions are deposited onto the WiMTiP surface using a micropipette at the desired volumes. Images of the sessile drops are captured, and the contact angles are extracted using the ImageJ plugin 'DropSnake'.[3] The liquid-vapor interfacial tension is measured using the pendant drop method, where the pendant drops are imaged using the same goniometer setup. The drops are suspended from a metal hub needle (25 gauge, Hamilton) connected to a 1 mL Hamilton glass syringe. The liquid-vapor interfacial tension is then extracted using the ImageJ plugin 'Pendent_Drop'.[4]

S.8 Hilbert-Huang Transformation. The Hilbert-Huang transformation consists of two steps [5]. The first step involves decomposing the solid-liquid interfacial tension γ_{SL} signal into a residual function γ_{SL}^{R} and an oscillating term γ_{SL}' using empirical mode decomposition (EMD)

$$\gamma_{SL}(t) = \gamma_{SL}^{R}(t) + \sum \gamma_{SL,i}'(t) \tag{S5}$$

The second term in Equation S5 denotes the summation of all the i-th intrinsic mode functions (IMFs) obtained during the EMD operation. Briefly, the local minima and maxima of the γ_{SL} signal are found and used to form the lower and upper envelopes. The mean of the envelopes

is subtracted from the original γ_{SL} signal to obtain the residual. These two processes are repeated until the residual meets the criterion of the IMF, and the resulting signal is the first IMF. This iterative process is repeated to extract other IMFs until no more IMFs can be further extracted, resulting in the final residual plotted in Figure S11 and S12 (Supporting Information). The residual is used to estimate the time mean of the γ_{SL} oscillation within a certain time window $[t_0, t_0 + \Delta t]$ (Table 1, first column) where

$$\overline{\gamma_{SL}^{R}} = \frac{1}{\Delta t}\int_{t_0}^{t_0+\Delta t} \gamma_{SL}^{R}(t)\, dt \quad \text{(S6)}$$

The oscillation amplitude $\sigma_{\gamma'_{SL}}$, on the other hand, can be simply estimated using the standard deviation of the γ_{SL} oscillation (Table 1, second column). However, this estimation may be biased by the slow undulation of the residual function. Hence, we used the next step in the Hilbert-Huang transformation, where the Hilbert transformation is applied to each of the IMFs but not the residual function. The transformed IMF signal $z_i(t)$ has the following form

$$z_i(t) = a_i(t)e^{j\theta_i(t)} \quad \text{(S7)}$$

where $a_i(t)$ is the instantaneous amplitude and $\theta_i(t)$ is the instantaneous phase. The instantaneous energy $EG_i(t)$ and instantaneous frequency $f_i(t)$ are defined as $|a_i(t)|^2$ and $d\theta_i(t)/dt$, respectively. One example of the time-frequency spectrum of one IMF is shown in Figure S14 (Supporting Information). The amplitude of the γ_{SL} oscillation is characterized by the total energy E, which is calculated by summing up all the instantaneous energies contained in all IMFs. The relative total energy REG is then calculated by normalizing with respect to the total energy of DI water. On the other hand, the expected frequency of the γ_{SL} oscillation f is calculated as the weighted average of the mean frequency of each IMF, using the total energy in each IMF as the weight. Mathematically,

$$EG = \sum_{i=1}^{m}(EG_i) = \sum_{i=1}^{m}\left(\int_{t=0}^{t} |a_i(t)|^2\ dt\right) \quad \text{(S8)}$$

$$REG = \frac{EG_k}{EG_{DI}} \quad \text{(S9)}$$

$$f = \frac{\sum_{i=1}^{m}\left(\int_{t=0}^{t}\frac{|a_i(t)|^2}{EG_i}\cdot\frac{d\theta_i(t)}{dt}\,dt\right)}{m} \quad \text{(S10)}$$

where EG_i denotes the energy of the solution k, m denotes the number of IMFs, EG_i denotes the total energy of the i-th IMF. By removing the residual for the energy and frequency calculation, we provide an unbiased quantification of the γ_{SL} oscillation.

S.9 Uncertainty Analysis. To evaluate the uncertainty of solid-liquid interfacial tension $E_{\gamma_{SL}}$ due to the *z* resolution, the following equation is invoked

$$E_{\gamma_{SL}}(\theta_1,\theta_2,\theta_3,\gamma_{LV}) = \sqrt{\frac{1}{n}\sum\left(\frac{\delta\gamma_{SL}}{\delta\theta_1}\Delta\theta_1\right)^2+\left(\frac{\delta\gamma_{SL}}{\delta\theta_3}\Delta\theta_3\right)^2+\left(\frac{\delta\gamma_{SL}}{\delta\theta_3}\Delta\theta_3\right)^2+\left(\frac{\delta\gamma_{SL}}{\delta\gamma_{LV}}\Delta\gamma_{LV}\right)^2} \quad \text{(S11)}$$

where $\Delta\theta_1$, $\Delta\theta_2$, $\Delta\theta_3$, and $\Delta\gamma_{LV}$ denotes the measurement uncertainty of θ_1, θ_2, θ_3, and γ_{LV} respectively. n denotes the number of radial profiles averaged in the azimuthal direction. The factor $\frac{1}{\sqrt{n}}$ takes into account the reduction of uncertainty due to averaging multiple radial profiles. Here $n = 3600$ as a 0.1° increment is used for the averaging. By substituting Equation S3 into Eqn. S4, one can obtain a function of γ_{SL} with respect to the 4 variables

$$\gamma_{SL} = \gamma_{LV}\left(\frac{\sin\theta_1 - \tan\theta_3\cos\theta_1}{\tan\theta_1\cos\theta_2 + \sin\theta_2}\right) \quad \text{(S12)}$$

To link $\Delta\theta_2$ and $\Delta\theta_3$ to Δz, the situation shown in Figure S15 (Supporting Information) is considered. We assume the Δz is manifested at the tip of the wetting ridge, thus making the fitted triangle increase in height. By denoting θ_2' and θ_3' as the two angles in the new triangle, the following relationships can be derived

$$\theta_2' = \tan^{-1}\frac{(z+\Delta z)\tan\theta_2}{z} \quad \text{(S13)}$$

$$\theta_3' = \tan^{-1}\frac{(z+\Delta z)\tan\theta_3}{z} \quad \text{(S14)}$$

We define the uncertainty as the difference between the new and old angles, i.e.,

$$\Delta\theta_2 = \theta_2' - \theta_2 = \tan^{-1}\frac{(z+\Delta z)\tan\theta_2}{z} - \theta_2 \qquad \text{(S15)}$$

$$\Delta\theta_3 = \theta_3' - \theta_3 = \tan^{-1}\frac{(z+\Delta z)\tan\theta_3}{z} - \theta_3 \qquad \text{(S16)}$$

The partial differentiation is carried out using MATLAB, and the uncertainty analysis results for various cases are presented in Table 1, third column.

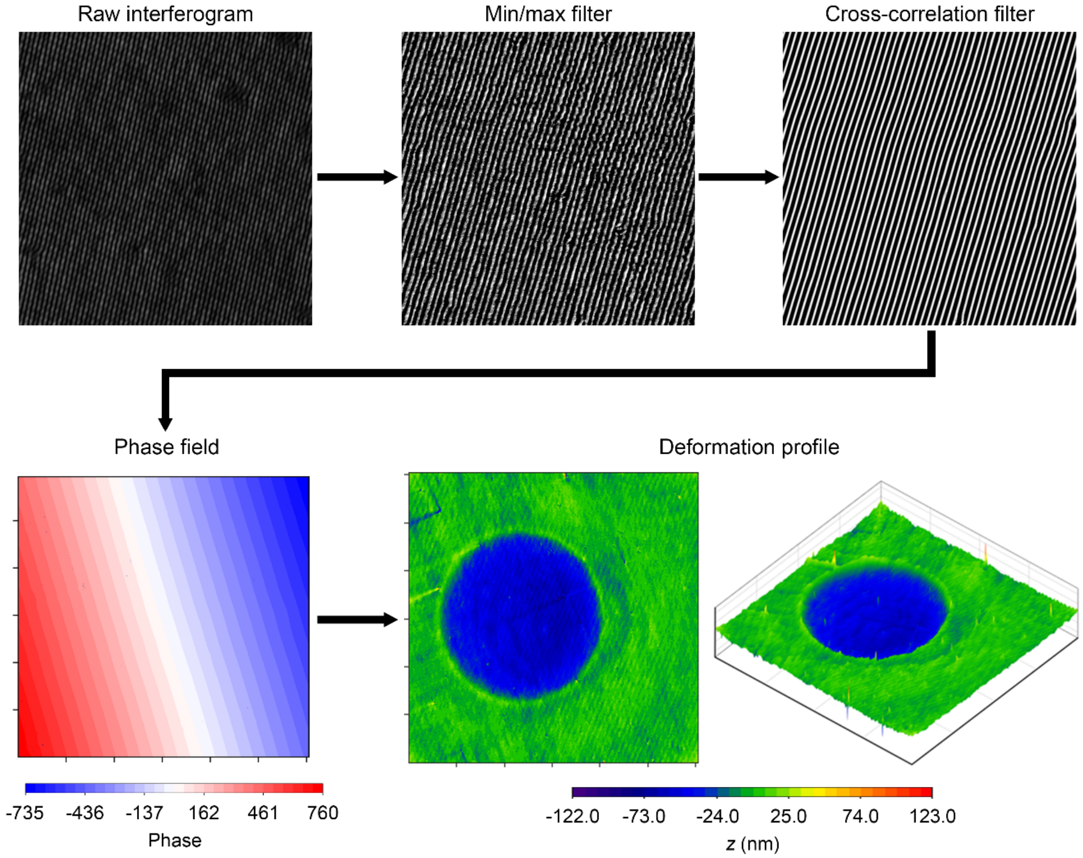

Figure S1. Data processing steps to extract the deformation profiles from raw interferograms obtained using the interferometric nanostrain sensor.

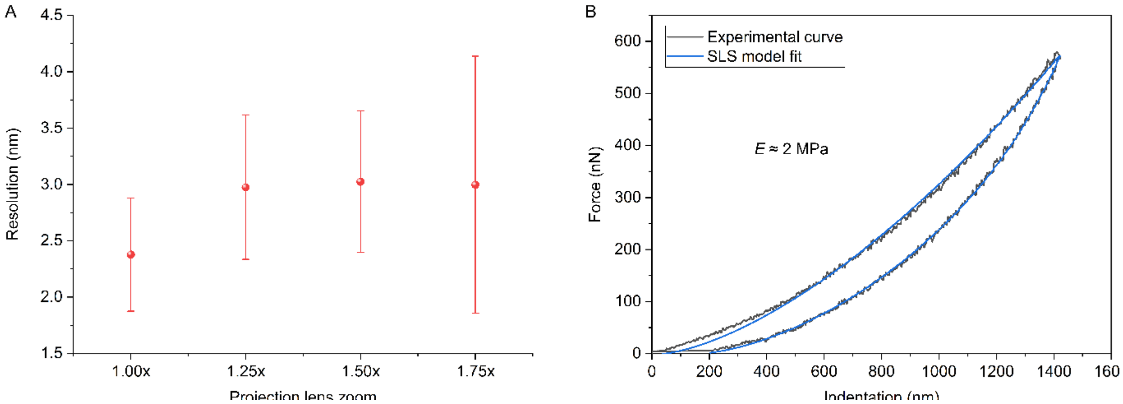

Figure S2. Characterization of the interferometric nanostrain sensor. (A) *Z* resolution of the interferometric nanostrain sensor with respect to the projection lens zoom. (B) The experimentally obtained force-indentation curve and the fitted standard linear solid (SLS) model with Ting's integral of the WiMTiP sensor. The estimated elastic modulus of the WiMTiP sensor is 2 MPa.

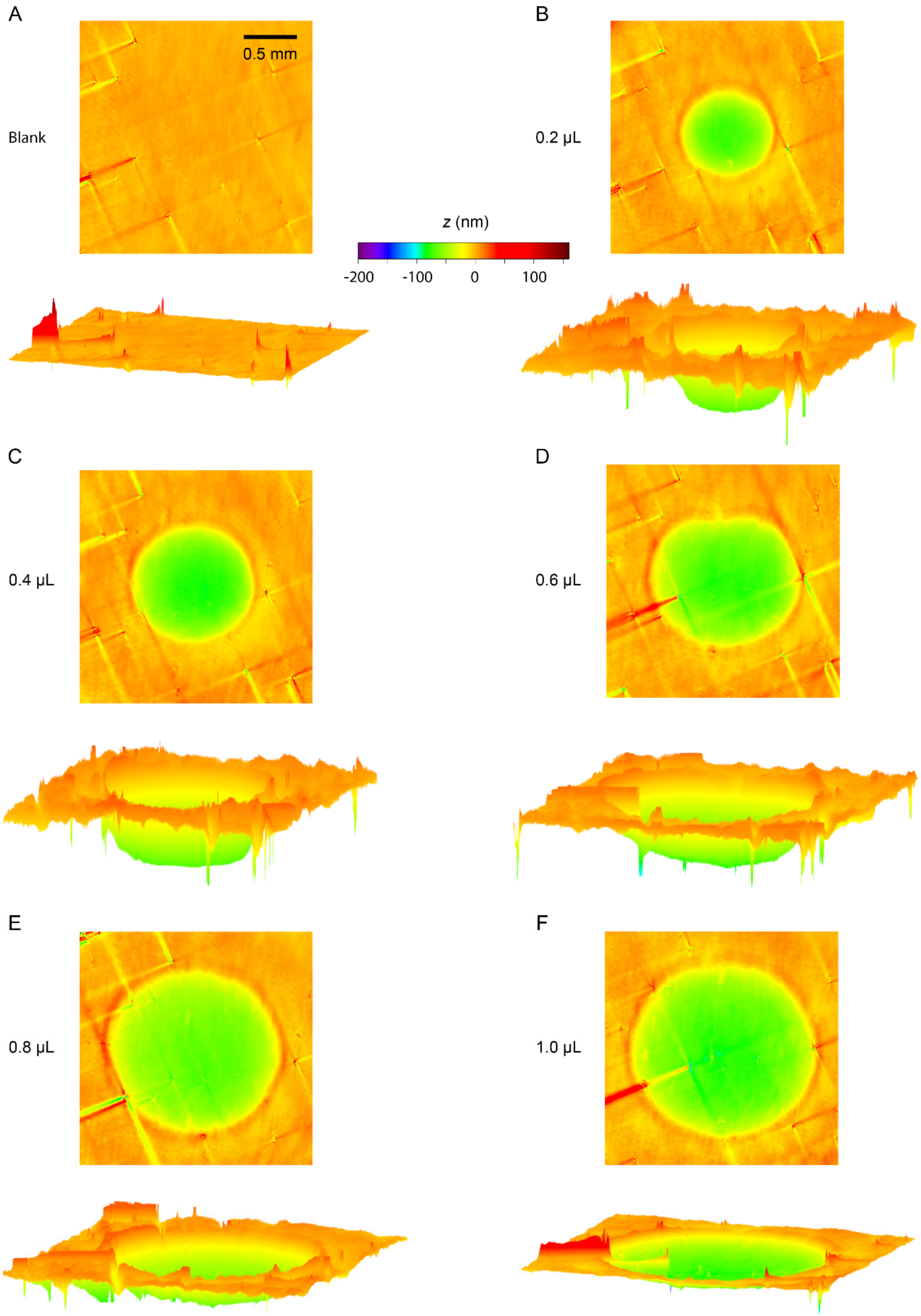

Figure S3. The 2D and 3D deformation profiles of (A) the undeformed WiMTiP sensor and sessile DI water drops of volumes (B) 0.2 μL, (C) 0.4 μL, (D) 0.6 μL, (E) 0.8 μL, and (F) 1.0 μL. Scale bar: 0.5 mm.

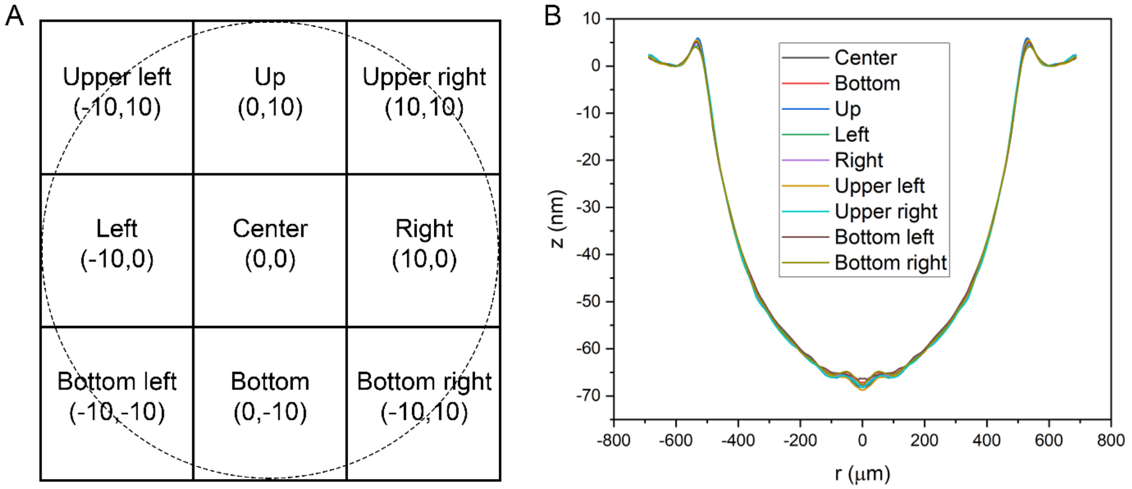

Figure S4. Center point analysis for dimple undulations. (A) Relative coordinates of the center point used for the calculation of the radial deformation profile. (B) Deformation profiles of a 0.6 μL sessile DI water drop when using the center points in (A). The undulations in the dimple are observed for all center points used.

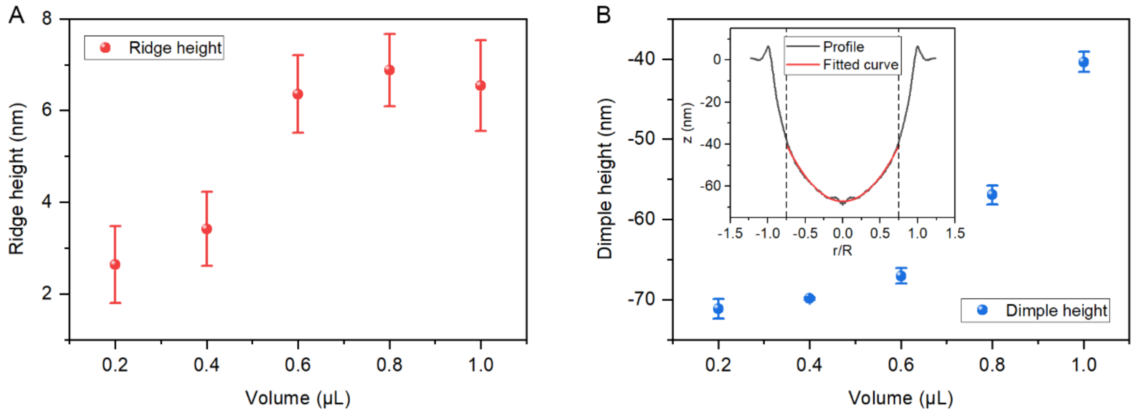

Figure S5. Characterization of the geometrical properties of the sessile DI water drops deformation profiles. The variation of the (A) ridge height and (B) dimple height with respect to the volume of the DI water drops. The inset shows an example of the normalized radial deformation profile of a 0.6 μL sessile DI water droplet (black curve) and the fitted polynomial curve (red curve) for the estimation of the dimple height. The dashed line represents the data range used for the fitting of the polynomial curve.

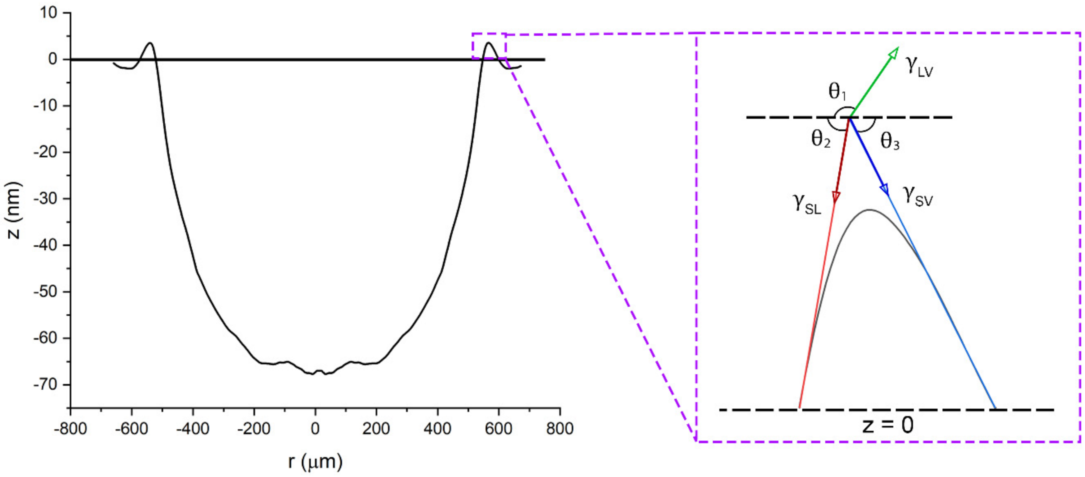

Figure S6. Curve fitting to obtain θ_2 and θ_3 for the extraction of the solid-liquid and solid-vapor interfacial tension. An example of a radial deformation profile of a sessile DI water drop on the WiMTiP sensor is shown. The inset shows the magnified view of the wetting ridge tip where two straight lines are fitted to the linear portion of the ridge, defined as 1/3 of the curve starting from $z = 0$ to the tip. The fitted lines are shown in red and blue for the left and right parts, respectively, of the wetting ridge. The intersection between the two fitted lines is found, and the angle relative to a horizontal line is calculated to obtain θ_2 and θ_3. θ_1 is obtained from the contact angle measurement.

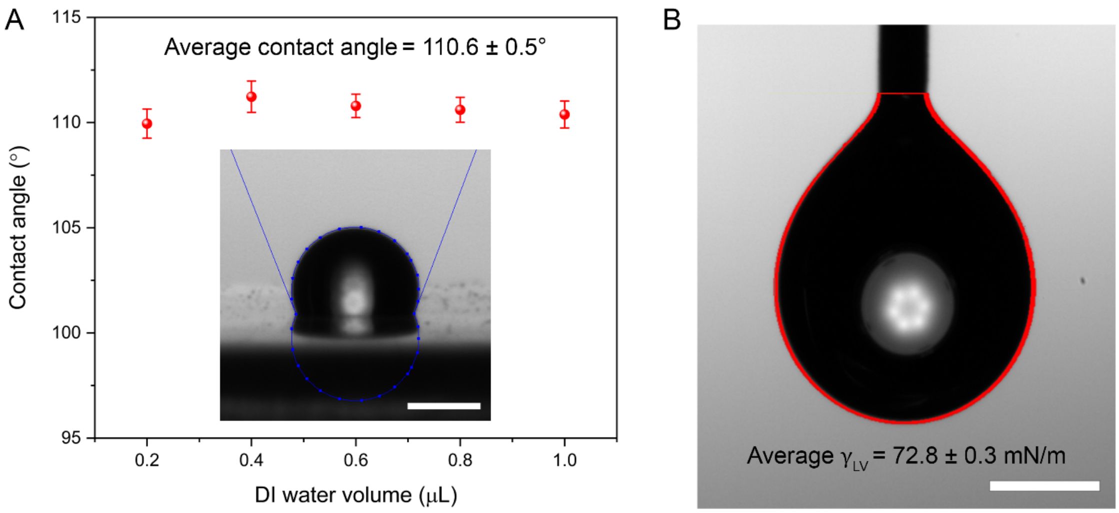

Figure S7. Estimation of the contact angle and liquid-vapor interfacial tension of DI water. (A) Contact angles of DI water drop of various volumes on the WiMTiP sensor surface. The inset image shows a representative analysis for the DI water contact angle using the ImageJ plugin 'Drop_snake'. (B) Pendant drop of DI water and the fitted pendant drop profile using the ImageJ plugin 'Pendent_Drop'. Standard deviations are calculated from 3 replicates. Scale bar: 1 mm.

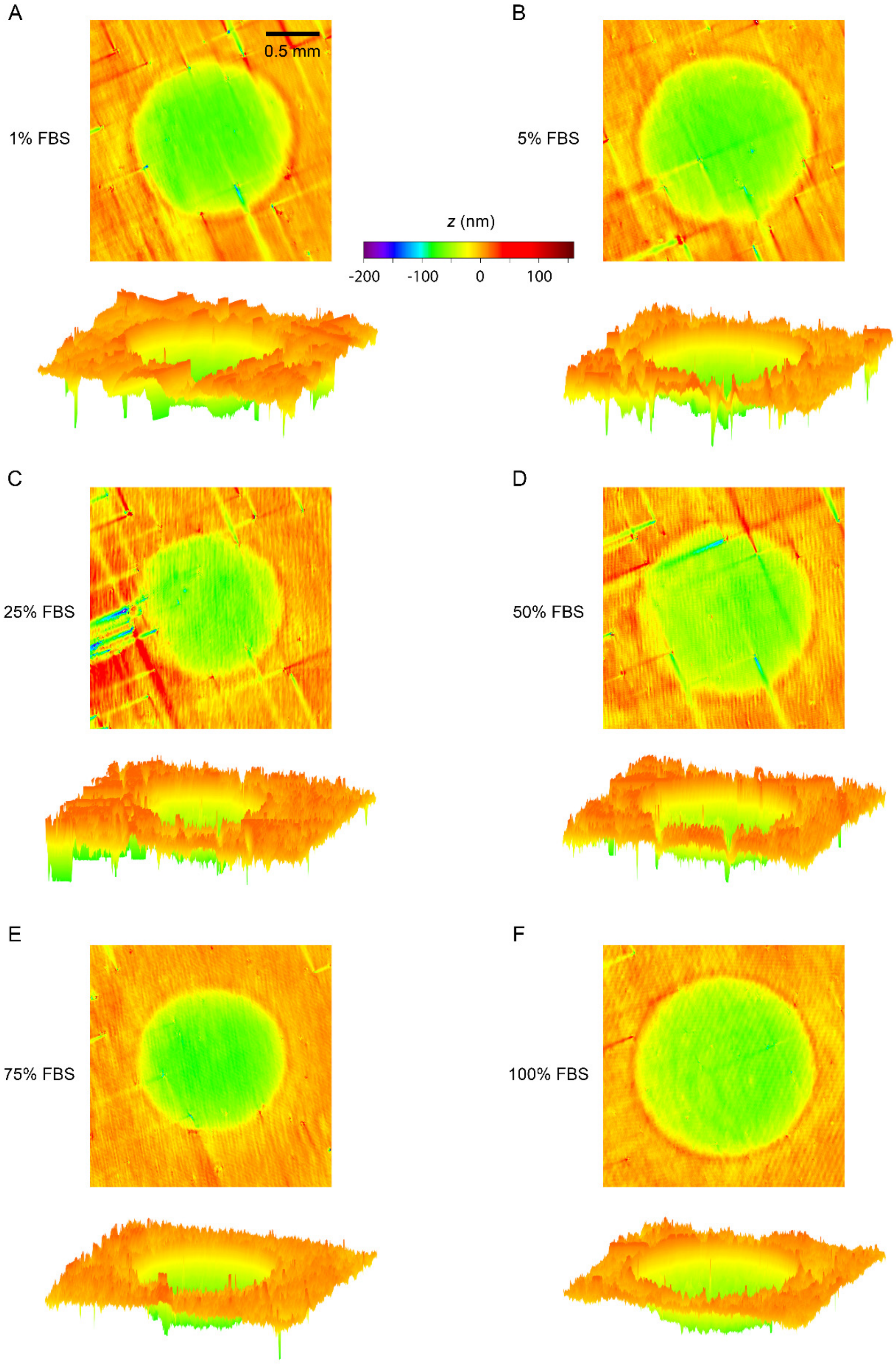

Figure S8. 2D and 3D deformation profiles of FBS sessile drops at (A) 1%, (B) 5%, (C) 25%, (D) 50%, (E) 75%, and (F) 100% concentration.

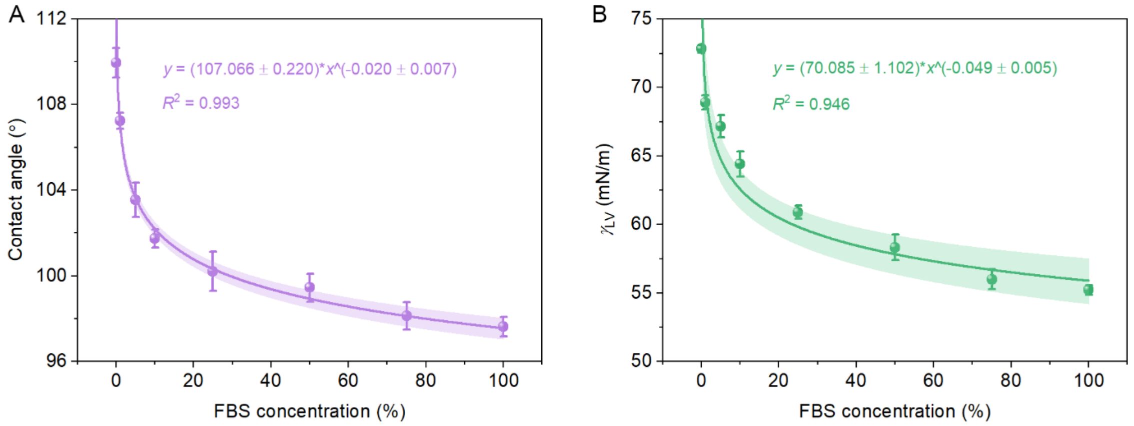

Figure S9. (A) Contact angle and (B) liquid-vapor interfacial tension of FBS solution with different concentrations. A power law is fitted to both variables and the corresponding fitted equation and adjusted R^2 are reported. Shaded areas represent the confidence band of the fitted curves.

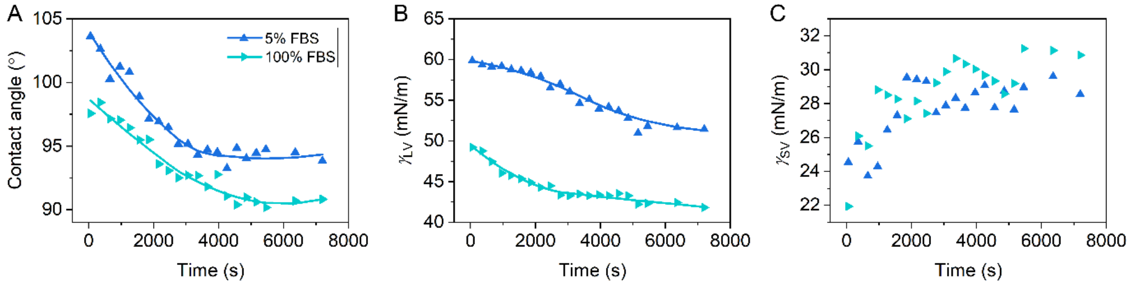

Figure S10. (A) Contact angle, (B) liquid-vapor interfacial tension, and (C) solid-vapor interfacial tension of 5% FBS and 100% FBS solutions over 2 hours. The solid lines represent the smooth curve calculated using the rloess (robust locally estimated scatterplot smoothing) regression method.

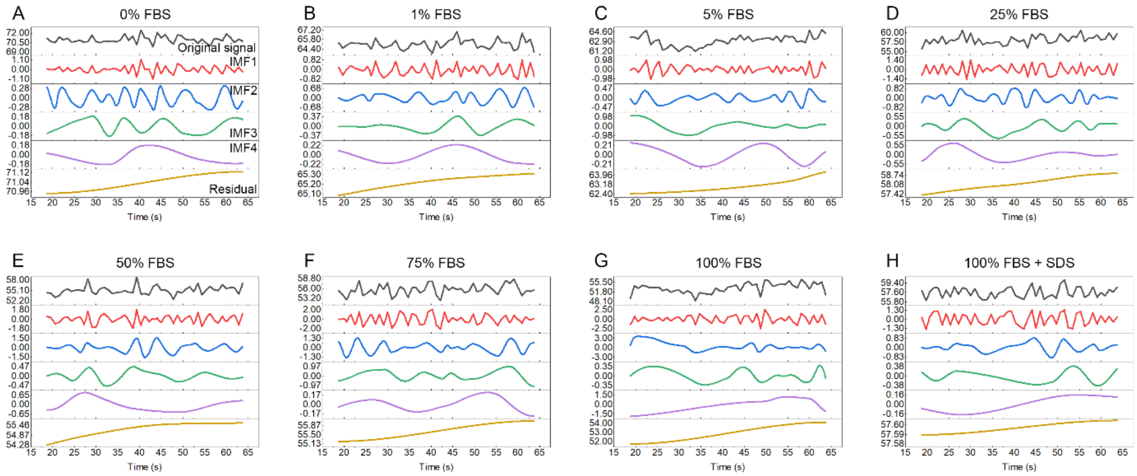

Figure S11. The original solid-liquid interfacial tension γ_{SL} oscillation signal, and the extracted IMFs and residual after applying the Hilbert-Huang transformation for (A) 0% (i.e. DI water), (B) 1%, (C) 5%, (D) 25%, (E) 50%, (F) 75%, (G) 100%, and (H) 100% FBS with the addition of SDS.

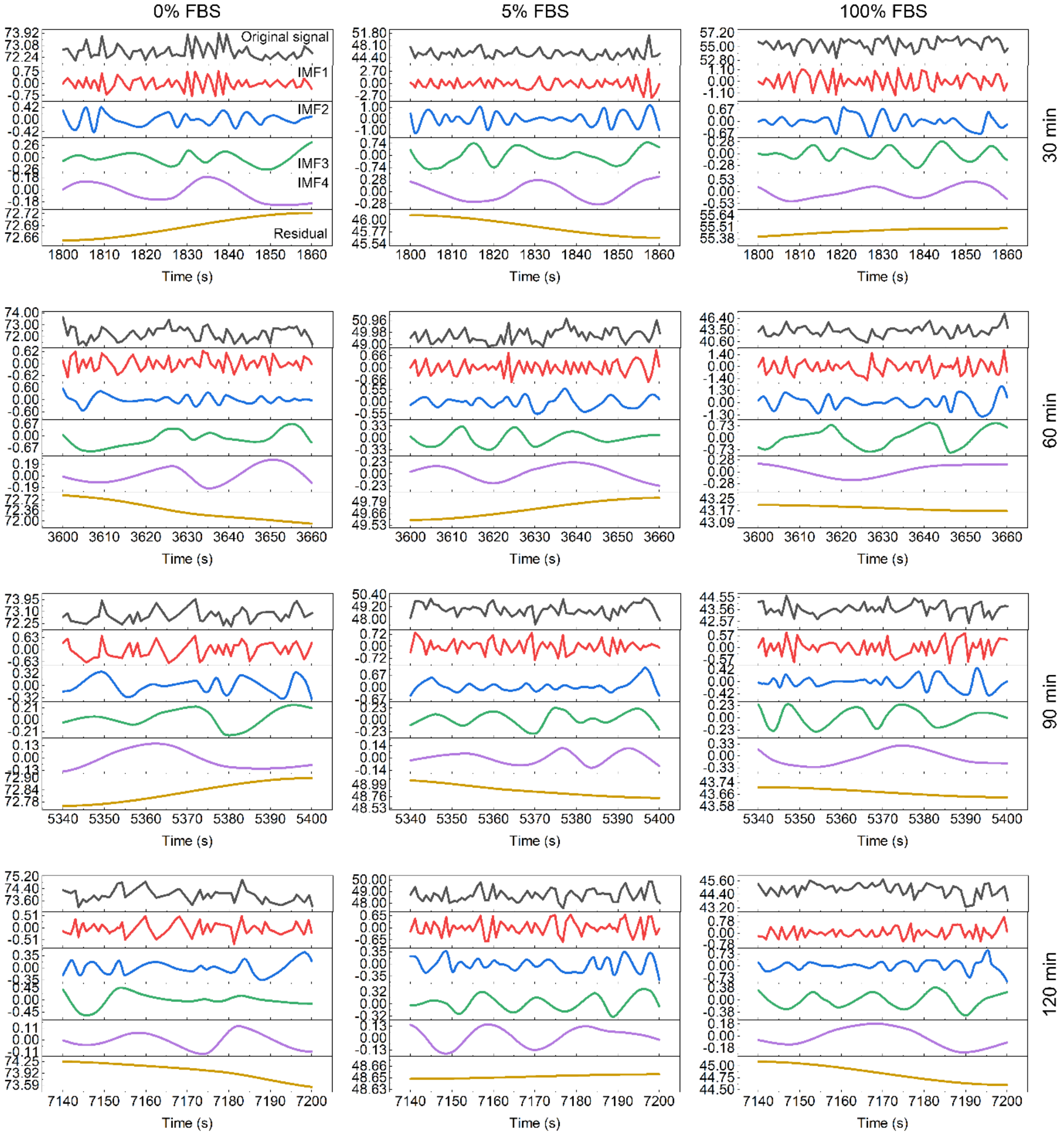

Figure S12. The original solid-liquid interfacial tension γ_{SL} oscillation signal, and the extracted IMFs and residual after applying the Hilbert-Huang transformation for 0% FBS (DI water), 5% FBS, and 100% FBS solution at various time points. The columns represent the concentration, whereas the rows denote the time points.

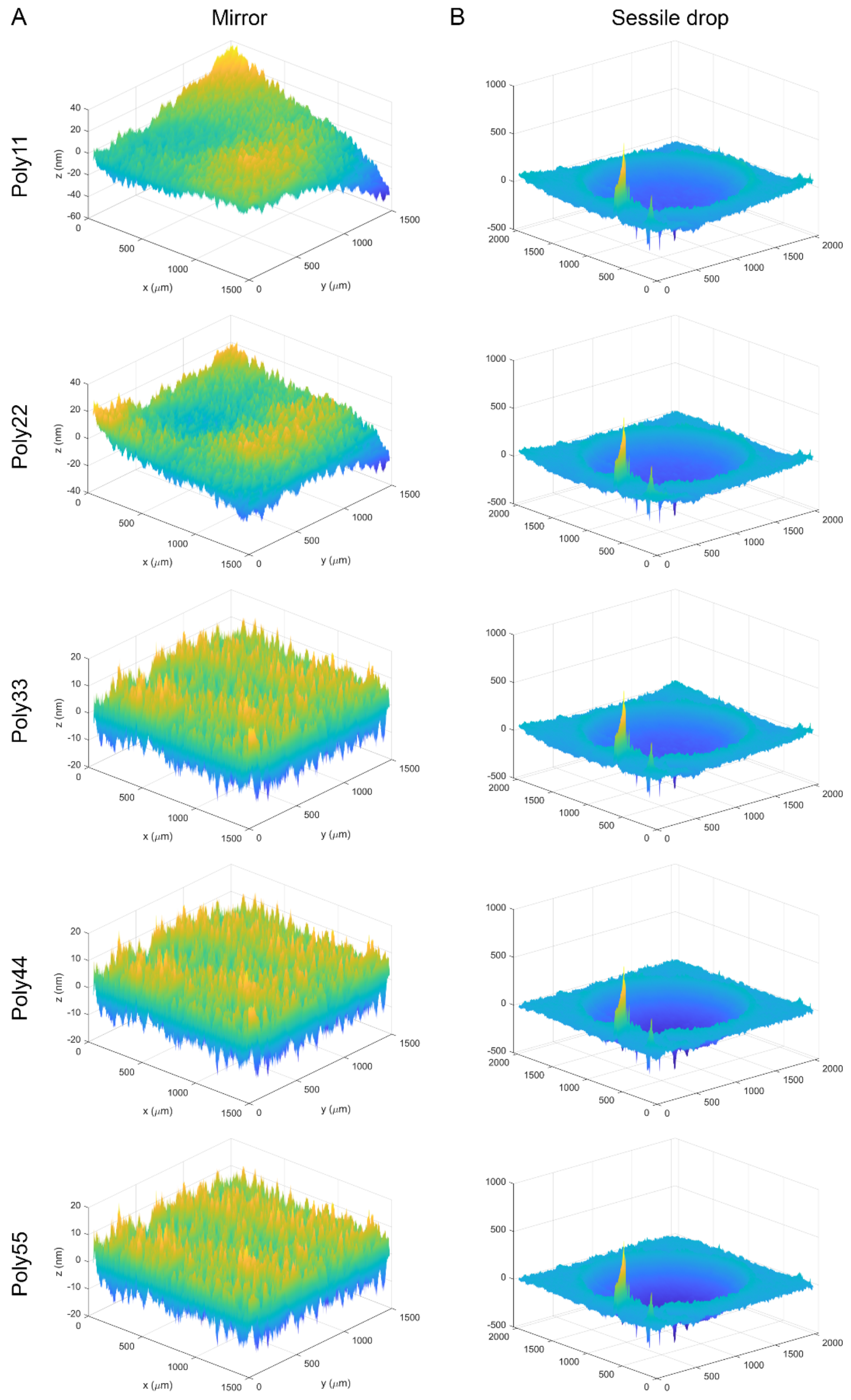

Figure S13. The deformation fields obtained using polynomial background correction of various orders for (A) a flat mirror (left column) and (B) a sessile drop on the WiMTiP (right column).

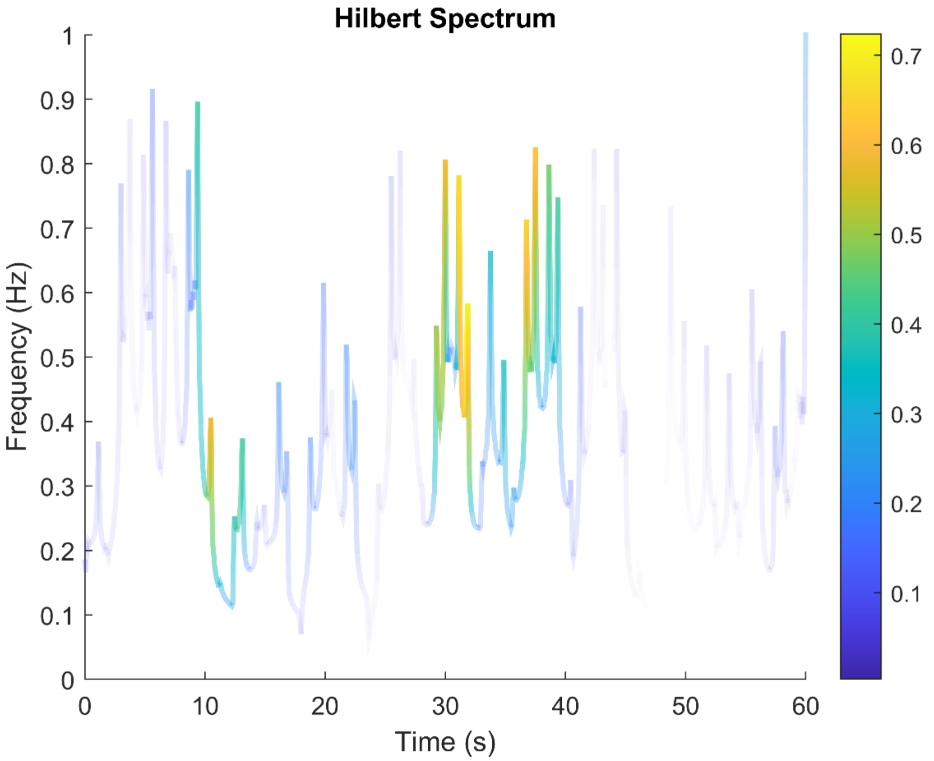

Figure S14. The Hilbert spectrum (i.e., instantaneous frequency vs time) for the first IMF component of the DI water γ_{SL} oscillation signal at $t_0 = 30$ min.

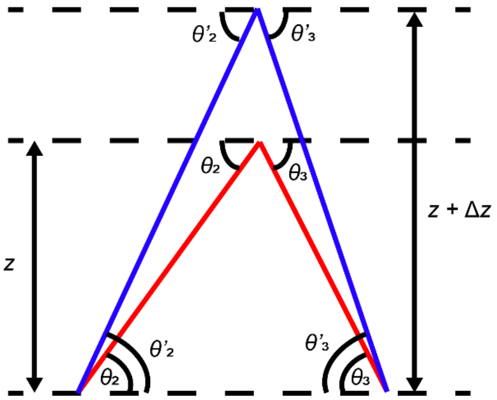

Figure S15. Geometrical considerations used for the uncertainty analysis of the solid-liquid interfacial tension.

Table S1: Dimension of competitive adsorption for each suspension used.

Solution	Dimension	Competing elements
100% Fetal bovine serum (FBS)	5	(1) Protein type (>1000 components)<br>(2) Affinities:<br>(3) Size:<br>(4) **C**onformation<br>(5) Ionic strength (e.g., ion concentrations)
100% FBS+SDS	4	(1) Protein type (>1000 components)<br>(3) Size:<br>(4) **C**onformation<br>(5) Ionic strength (e.g., ion concentrations)
5% Denatured FBS	3	(1) Protein type (>1000 components)<br>(3) Size:<br>(5) Ionic strength (e.g., ion concentrations)
25% Bovine serum albumin (BSA)	2	(2) Affinities:<br>(4) **C**onformation
Polystyrene nanoparticles (NP)	0	

Table S2: Typical composition of FBS and the molecular mass and concentration of each composition.

Category	Components	Molecular mass (Da)	Concentration	Unit
Proteins	Serum albumin	66500	25.00	mg/mL
	Alpha-1-antiproteinase	52000	10.51	mg/mL
	Plasminogen	92000	8.51	mg/mL
	Cone cGMP-specific 3′,5′-cyclic phosphodiesterase alpha-subunit	99100	8.11	mg/mL
	Lactoperoxidase	78000	8.11	mg/mL
	NADH-ubiquinone oxidoreductase 75	76000	6.91	mg/mL
	Alpha-2-HS-glycoprotein	49000	6.91	mg/mL
	Kininogen, LMW II	70000	6.25	mg/mL
	Integrin beta-1	88000	6.25	mg/mL
	Prothrombin	72000	5.85	mg/mL
	Apolipoprotein A-I	28100	5.05	mg/mL
	Antithrombin-III	58000	5.05	mg/mL
	Beta-2-glycoprotein I	48000	4.65	mg/mL
	Alpha-2-antiplasmin	67000	4.26	mg/mL
	Alpha-1-1-microglobulin and inter alpha-trypsin inhibitor light chain	26000	3.86	mg/mL
	Alpha 1 antichymotrypsin	60500	3.06	mg/mL
	Transferrin	80000	2.00	mg/dL
	Alkaline phosphatase	86000	351.00	U/L
	Amylase	52000	19.00	U/L
	Lactic dehydrogenase	140000	1315.00	U/L
	Glutamic oxaloacetic transaminase	92000	41.00	UI/L
Hormones	Adrenocorticotropic hormone	2600	1.00	pg/mL
	Luteinizing hormone	30000	0.02	U/L
	Prolactin	23000	0.60	ng/mL
	Parathormone	9500	95.00	pg/mL
	Progesterone	317	0.10	ng/mL
	Follicle-stimulating hormone	30000	0.05	mU/mL
	Insulin	5808	0.43	mU/mL
	Estradiol	272	29.00	pg/mL
	Parathyroid hormone	3334	70.33	pg/mL
Fatty acids and lipids	Low-density lipoprotein	2930000	11.67	mg/mL
	High-density lipoprotein	267000	10.00	mg/mL
Vitamins	Vitamin A	286	0.20	mg/L
	Vitamin B12	1355	245.33	pg/mL
	Vitamin C	176	0.06	mg/L
	Vitamin D3	384	13.27	ng/mL

	Vitamin E	430	0.43	mg/L
	Folic acids	441	4.53	ng/ml
Minerals	Sodium	23	130.00	mmol/L
	Potassium	39	9.70	ng/mL
	Magnesium	24	2.80	mg/dL
	Iron	56	169.00	µg/dL
	Calcium	40	12.63	mg/dL
Others	Bilirubin	585	0.23	mg/dL
	Chlorine	35	105.67	meq/L

These data are adapted from references [6-10].

Reference

[1] Zhang, C.; Miorini, R.; Katz, J., Integrating Mach–Zehnder interferometry with TPIV to measure the time-resolved deformation of a compliant wall along with the 3D velocity field in a turbulent channel flow, *Exp. Fluids* **2015,** *56* (11), 203.

[2] Efremov, Y. M.; Wang, W. H.; Hardy, S. D.; Geahlen, R. L.; Raman, A., Measuring nanoscale viscoelastic parameters of cells directly from AFM force-displacement curves, *Sci. Rep.* **2017,** *7* (1), 1541.

[3] Stalder, A. F.; Melchior, T.; Müller, M.; Sage, D.; Blu, T.; Unser, M., Low-bond axisymmetric drop shape analysis for surface tension and contact angle measurements of sessile drops, *Colloids Surf. Physicochem. Eng. Aspects* **2010,** *364* (1-3), 72-81.

[4] Daerr, A.; Mogne, A., Pendent_Drop: An ImageJ Plugin to Measure the Surface Tension from an Image of a Pendent Drop, *Journal of Open Research Software* **2016,** *4* (1).

[5] Huang, N. E., *Hilbert-Huang transform and its applications*. World Scientific: **2014**; Vol. 16.

[6] Efstratiou, M.; Christy, J. R. E.; Bonn, D.; Sefiane, K., Transition from Dendritic to Cell-like Crystalline Structures in Drying Droplets of Fetal Bovine Serum under the Influence of Temperature, *Langmuir* **2022,** *38* (14), 4321-4331.

[7] Lee, D. Y.; Lee, S. Y.; Yun, S. H.; Jeong, J. W.; Kim, J. H.; Kim, H. W.; Choi, J. S.; Kim, G. D.; Joo, S. T.; Choi, I.; Hur, S. J., Review of the Current Research on Fetal Bovine Serum and the Development of Cultured Meat, *Food Sci Anim Resour* **2022,** *42* (5), 775-799.

[8] Stival, A. C. S.; da Silva, A. C. G.; Valadares, M. C., Qualitative and quantitative evaluation of Fetal Bovine Serum composition: toward ethical and best quality in vitro science, *NAM Journal* **2025,** *1*, 100047.

[9] Sakulkhu, U.; Mahmoudi, M.; Maurizi, L.; Coullerez, G.; Hofmann-Amtenbrink, M.; Vries, M.; Motazacker, M.; Rezaee, F.; Hofmann, H., Significance of surface charge and shell material of superparamagnetic iron oxide nanoparticle (SPION) based core/shell nanoparticles on the composition of the protein corona, *Biomaterials Science* **2015,** *3* (2), 265-278.

[10] Zheng, X.; Baker, H.; Hancock, W. S.; Fawaz, F.; McCaman, M.; Pungor Jr., E., Proteomic Analysis for the Assessment of Different Lots of Fetal Bovine Serum as a Raw Material for Cell Culture. Part IV. Application of Proteomics to the Manufacture of Biological Drugs, *Biotechnology Progress* **2006,** *22* (5), 1294-1300.
